# The cervical lymph node contributes to peripheral inflammation related to Parkinson’s disease

**DOI:** 10.1186/s12974-023-02770-5

**Published:** 2023-04-10

**Authors:** Zongran Liu, Yang Huang, Xuejing Wang, Jia-Yi Li, Can Zhang, Ying Yang, Jing Zhang

**Affiliations:** 1 Department of Pathology, The First Affiliated Hospital of Zhejiang University School of Medicine, Hangzhou, 310002 Zhejiang China; 2 Department of Pathology, Peking University Health Science Center, Beijing, 100191 China; 3 Department of Neurology, The First Affiliated Hospital of Zhengzhou University, Zhengzhou, 450052 Henan China; 4 Institute of Parkinson and Movement Disorder, Zhengzhou University, Zhengzhou, 450052 Henan China; 5 Neural Plasticity and Repair Unit, Department of Experimental Medical Science, Wallenberg Neuroscience Center, Lund University, Lund, Sweden; 6 Institute of Health Sciences, China Medical University, Shenyang, 110122 Liaoning China; 7 National Human Brain Bank for Health and Disease, Zhejiang University, Hangzhou, 310002 Zhejiang China

**Keywords:** Cervical lymph node, Parkinson’s disease, α-Synuclein, Endoplasmic reticulum stress, Macrophage, Inflammation

## Abstract

**Background:**

Peripheral inflammation is an important feature of Parkinson’s disease (PD). However, if and how CNS pathology is involved in the peripheral inflammation in PD remains to be fully investigated. Recently, the existence of meningeal lymphatics and its involvement in draining cerebral spinal fluid (CSF) to the cervical lymph node has been discovered. It is known that meningeal lymphatic dysfunction exists in idiopathic PD. The deep cervical lymph node (dCLN) substantially contributes to the drainage of the meningeal lymphatics. In addition, one of the lymphatics draining components, CSF, contains abundant α-synuclein (α-syn), a protein critically involved in PD pathogenesis and neuroinflammation. Thus, we began with exploring the possible structural and functional alterations of the dCLN in a PD mouse model (A53T mice) and investigated the role of pathological α-syn in peripheral inflammation and its potential underlying molecular mechanisms.

**Methods:**

In this study, the transgenic mice (prnp-SNCA*A53T) which specifically overexpressed A53T mutant α-syn in CNS were employed as the PD animal model. Immunofluorescent and Hematoxylin and eosin staining were used to evaluate structure of dCLN. Inflammation in dCLNs as well as in bone-marrow-derived macrophages (BMDMs) was assessed quantitatively by measuring the mRNA and protein levels of typical inflammatory cytokines (including IL-1β, IL-6 and TNF-α). Intra-cisterna magna injection, flow cytometric sorting and electrochemiluminescence immunoassays were applied to investigate the lymphatic drainage of α-syn from the CNS. RNA-seq and Western blot were used to explore how pathological α-syn mediated the inflammation in PD mice.

**Results:**

The results unequivocally revealed substantially enlarged dCLNs, along with slow lymphatic flow, and increased inflammation in the dCLNs of A53T mice. Oligomeric α-syn drained from CSF potently activated macrophages in the dCLN via endoplasmic reticulum (ER) stress. Notably, inhibition of ER stress effectively suppressed peripheral inflammation in PD mice.

**Conclusions:**

Our findings indicate that lymph node enlargement is closely related to macrophage activation, induced by meningeal lymphatics draining oligomeric α-syn, and contributes to the peripheral inflammation in PD. In addition, ER stress is a potential therapeutic target to ameliorate PD pathogenesis.

**Supplementary Information:**

The online version contains supplementary material available at 10.1186/s12974-023-02770-5.

## Background

Parkinson’s disease (PD), clinically characterized by motor and non-motor symptoms, is defined pathologically by the presence of Lewy bodies in surviving neurons [1, 2]. α-Synuclein (α-syn), the major component of Lewy bodies, is considered one of the most important players in PD pathogenesis [3–5], although many other mechanisms, including oxidative stress, mitochondrial failure, and neuroinflammation are also involved [6]. α-Syn in the central nervous system (CNS) is mainly an intracellular protein, and was initially thought to be primarily located in the nucleus and synapses [7–9]. Later studies have identified α-syn in other cellular locations, including mitochondria [2, 6], as well as several secreted forms, e.g., aggregated α-syn, in the cerebral spinal fluid (CSF) [10].

The central role of α-syn in PD pathogenesis relates to its uncontrolled aggregation from monomers to oligomers before forming fibrils in the Lewy bodies [11]. Physiologically, a delicate balance of various α-syn forms is maintained mainly via intracellular protein metabolism, including the ubiquitin-proteasome and the lysosomal autophagy systems [12, 13]. More recently, increasing evidence indicates that extracellular secretion of α-syn either as free forms or contained in the extracellular vesicles, e.g., exosomes [14, 15], is also critical to the α-syn homeostasis in the CNS.

Disruption of the α-syn homeostasis triggers multiple cellular processes, among which inflammation is being investigated most vigorously in recent years [6, 16]. Apart from neuroinflammation manifested by microglial activation, increased inflammatory cytokines and infiltration of immune cells in the CNS [6, 17–19], peripheral inflammation, reflected by increased inflammatory cytokines and abnormal hyperactivation of immune cells, has also been observed in PD patients [20–23]. Clearly, both CNS and peripheral inflammation could aggravate the development of PD [24–26]. However, the relation between these two types of inflammation remains to be characterized.

CNS α-syn is well-known to be transported to the blood via the blood-brain barrier (BBB) [27–29]. The discovery of the meningeal lymphatics network recently has provided another possible route for the communication of α-syn between the CNS and the peripheral system in PD [30, 31]. It has been demonstrated that meningeal lymphatics can drain CSF and its content into the cervical lymph node (CLN), where normal and potentially harmful contents in CSF are filtered or neutralized [32–34]. The meningeal lymphatic drainage of some neurodegenerative disease-related proteins, including tau, amyloid beta and α-syn, has been investigated in animal models and autopsy specimens [35–37]. In the lymphatic system, a key component is the lymph node, which typically enlarges and becomes painful when inflammation occurs [38–40]. Whether the harmful species like aggregated α-syn in the CNS were involved in the peripheral inflammation in PD or related disorders through meningeal lymphatic drainage is largely unknown.

In this study, we explored the possible structural and functional alterations in the dCLN in a PD animal model, prnp-SNCA*A53T mice, which overexpress α-syn specifically in the CNS, with defined pathology evolving gradually in various brain regions [41]. We also tested the hypothesis that pathological α-syn aggregates in CSF could activate macrophages in the dCLN of A53T mice and investigated the role of pathological α-syn in peripheral inflammation and its potential underlying molecular mechanisms.

## Methods

### Animals

Mice were maintained and fed in a barrier environment in the Department of Laboratory Animal Science, Peking University Health Science Center (room temperature 18–22 °C, humidity 30–50%, well-ventilated, a 12-h light–dark cycle). Male and female mice were purchased from the Jackson Laboratory or Department of Laboratory Animal Science, Peking University Health Science Center. Strains used are C57Bl/6J (C57; JAX: 000664) and B6; C3-Tg (Prnp-SNCA*A53T)83Vle/J (A53T; JAX:004479).

All experiments were carried out on male mice at 8-month age. For A53T mice, age-matched B6C3F1/J mice (wild type, WT) were selected as the control group. Mice in the same experimental group were randomly selected from different cages. For TUDCA treatment, 7-month-old A53T mice were intraperitoneally injected with TUDCA at a dose of 100 mg/kg/day for 1 month. Additional details related to the mice utilized in the study are listed in Additional file 1: Table S1. All animal procedures were approved in accordance with the Chinese Guidelines for the ethical review of laboratory animal welfare (LA2020056).

### Intracisterna magna (i.c.m.) injection

Mice were anaesthetized by intraperitoneal (i.p.) injection of Tribromoethanol (Avertin, 12.5 mg/mL, 0.2 mL/10 g body weight) and fixed in a stereotaxic frame. The skin of the neck was incised and the muscle layers were retracted to expose the posterior atlanto-occipital membrane. A 10 μL Hamilton syringe adapted with 33-gauge single injector was penetrated into the cisterna magna, and 5 μL Alexa Flour 647-labelled α-syn (1 mg/mL), or 4 μL Qdot 605 were infused into the subarachnoid space at 1 μL/min with a syringe pump (Harvard Apparatus). The syringe was left in place for 2 min to prevent backflow of CSF. Subsequently, the neck skin was then sutured, after which the mice were subcutaneously injected with Flunixin (5 mg/kg) and allowed to recover on a heat pad until fully awake, and later sacrificed for the tissue collection.

### Tissue collection and processing

Mice were anaesthetized by intraperitoneal (i.p.) injection of Tribromoethanol (Avertin, 12.5 mg/mL) and transcardially perfused with ice-cold PBS.

For cervical lymph node collection, the neck skin was longitudinally incised superior to the clavicle and the sternocleidomastoid muscles were retracted, so that the deep cervical lymph node (dCLN) could be harvested by forceps.

For brain collection, after lymph node collection, the mice were decapitated above the shoulders. Then, the top of the skull was removed with surgical curved scissors by cutting clockwise, and the brain was collected for further study.

For whole-mount meninges collection, the skullcap was fixed ahead, and then the meninges were carefully dissected from the skullcaps with forceps and kept in PBS at 4 °C for further use.

The lymph nodes and brain were placed in 4% paraformaldehyde (PFA) at 4 °C for 24 h and then transferred to 30% sucrose for 24 h. After dehydration, lymph nodes and brain were embedded in OCT and sliced into 12-μm-thick sections (for immunostaining) or 6-μm-thick sections (for hematoxylin and eosin staining) by Cryostats microtome (Leica).

### Intravital microscopy of the deep cervical lymph node

Mice were anaesthetized by i.p. injection of Tribromoethanol (Avertin, 12.5 mg/mL) and fixed in a custom-made pad. The neck skin was longitudinally incised superior to the clavicle and the sternocleidomastoid muscles were retracted, so that the deep cervical lymph node (dCLN) could be exposed under the objectives. Then, mice were discharged and injected intracisterna magna (i.c.m) with Qdot 605. After re-fixing onto the pad, the signal of Qdot 605 in dCLN was recorded every 30 s for 80 min by Leica TCS SP5 confocal system (Leica Microsystems) with a HCX PL FLUOTAR 5.0 × 0.15 dry objective.

### Immunofluorescence

The tissue slices or whole-mount meninges were rinsed with PBS for 10 min and treated by blocking solution (1% BSA, 0.4% Triton X-100 and 4% goat serum in PBS) for 1 h at room temperature, followed by incubation with appropriate dilutions of primary antibodies: anti-LYVE-1 (eBioscience, ALY7, 1:200), anti-CD169 (Abcam, 3D6.112, 1:100), anti-α-synuclein (Abcam, MJFR-1, 1:250), anti-podoplanin (eBioscience, 1:100), anti-CD11b (Abcam, M1/70, 1:100), anti-B220 (eBioscience, RA3-6B2, 1:100), anti-CD3e (eBioscience, 145-2C11, 1:100), anti-F4/80 (eBioscience, BM8, 1:200), anti-GRP78 (Abcam, ab21685, 1:200), anti-IL-1β (Santa cruz, E7-2-hIL1β, 1:50), anti-IL-6 (Cell signaling technology, D5W4V, 1:200), and anti-TNF-α (Abcam, EPR19147, 1:1000) in blocking solution overnight at 4 °C. Then, the tissue slices or whole-mount meninges were washed with PBS for three times at room temperature and incubated with the appropriate dilutions of secondary antibodies including goat Alexa Fluor 488, 594 or 647 anti-rat, -rabbit, -mouse, -Armenian hamster or -Syrian hamster IgG antibodies (Abcam, 1:500) for 1 h at room temperature in PBS containing 0.3% of Triton X-100. After staining by DAPI for 5 min, the tissue slices or whole-mount meninges were rinsed with PBS three times for 5 min and covered by coverslips.

For cell immunofluorescence, RAW 264.7 cells were plated on microscopy grade petri dishes (Jet biofilm, BDD002035) at a density of 1 × 10^6^/mL and cultured with 500 nM α-syn for 12 h. Cells were incubated with the primary antibodies including GRP78 (1:200, Abcam, ab21685) and α-synuclein (1:200, Abcam, ab80627) overnight at 4 °C. Then, cells were incubated with secondary antibodies including goat Alexa Fluor 488 anti-mouse IgG antibodies (Abcam, 1:500) and goat Alexa Fluor 555 anti-rabbit IgG antibodies for 1 h at room temperature.

Images of tissue slices and cells were captured by a Zeiss LSM700 confocal microscope (Carl Zeiss) equipped with ZEN 3.0 software (Carl Zeiss) and acquired at a resolution of 2048 × 2048 pixels with air objectives: Plan-Apochromat 10×/0.45 numerical aperture (NA) M27 and Plan-Apochromat 20×/0.8 NA M27. Images of the whole-mount meninges were taken by Leica TCS SP8 confocal system (Leica Microsystems) with objectives and merged by LSA AF software.

### Staining and counting of tyrosine hydroxylase positive neurons

Immunohistochemistry (IHC) staining of tyrosine hydroxylase (TH) was performed on the brain sections from WT and A53T mice treated with vehicle or TUDCA. The right brain hemispheres were serially cut in the coronal plane at a thickness of 10 μm and collected 12 serial sections at 10 intervals (100 μm). The total number of TH^+^ neurons in each brain was used for comparison.

### Western blotting

Proteins were extracted from dCLN, brain and BMDM using cell lysis buffer (RIPA, HARVEYBIO, C1503) containing protease inhibitor cocktail (Sigma) and phosphatase inhibitor PhosSTOP™ (Roche, 4906837001). BCA Protein Assay Kit (Applygen, P1511) was applied to determining the protein concentration of dCLN, brain and BMDM according to the manufacturer’s instruction. A total of 30 μg dCLN, brain and BMDM lysates were loaded to 10%- or 12%-PAGE gels for electrophoresis. Separated proteins were transferred to the PVDF membrane (Merck Millipore, 4515). Then, the membranes were blocked in TBST buffer (Applygen, B1009) containing 5% BSA (Amresco, A-0332) for 1 h at room temperature and incubated with diluted primary antibody overnight at 4 °C. Antibodies used included IL-1β (1:200, Santa cruz, sc-32294), IL-6 (1:1000, Cell signaling technology, 12912), TNF-α (1:1000, Abcam, ab183218), p-IRE1α (1:1000, Abcam, ab124945), IRE1α (1:1000, Cell signaling technology, 3294), GRP78 (1:1000, Abcam, ab21685), ATF-6 (1:100, Santa cruz, sc-166659), β-actin (1:1000, Zsgb-bio, TA-09), ATF-4 (1:1000, Cell signaling technology, 11815S), sXBP-1 (1:1000, Biolegend, 658802), p-eIF2α (1:1000, Cell signaling technology, 3398S), eIF2α (1:1000, Cell signaling technology, 5324S), and α-synuclein (1:5000, Abcam, ab138501). The membranes were washed three times with TBST buffer on the second day, followed by incubation with secondary antibody for 1 h at RT. Protein bands were developed by enhanced chemiluminescence regents (Millipore, WBKLS0100) and detected by Chemidoc™ Imaging System (Bio-Rad, Hercules CA, USA).

### Real‑time quantitative PCR

Total RNA was isolated from dCLNs and BMDMs by TRIZOL™ reagent (Invitrogen, 15596018) according to the manufacturer’s instruction. Following the extraction of RNA, cDNA was reverse transcribed from 2 mg total RNA by RT Master Mix Kit with gDNase (MCE, HY-K0511). The mRNA level of target gene in acquired cDNA was determined by real-time PCR with PowerUp SYBR® Green Master Mix (applied biosystem, A25742). *Actb* was used as the internal reference for normalization. The primers used are presented in Table 1. All results were analyzed using 2^−△△Ct^ and were presented as mean ± SEM.

**Table 1:** Primers used in qPCR

Gene	Forward primer (5′ to 3′)	Reverse primer (5′ to 3′)
*Il1b*	TGGACCTTCCAGGATGAGGACA	GTTCATCTCGGAGCCTGTAGTG
*Il6*	TACCACTTCACAAGTCGGAGGC	CTGCAAGTGCATCATCGTTGTTC
*Tnf*	GGTGCCTATGTCTCAGCCTCTT	GCCATAGAACTGATGAGAGGGAG
*Actb*	CATTGCTGACAGGATGCAGAAGG	TGCTGGAAGGTGGACAGTGAGG
*Ern1*	GGCTACCATTATCCTGAGCACC	CTCCTTCTGGAACTGTTGGTGC
*Xbp1*	TGGACTCTGACACTGTTGCCTC	TAGACCTCTGGGAGTTCCTCCA
*Atf6*	GTCCAAAGCGAAGAGCTGTCTG	AGAGATGCCTCCTCTGATTGGC
*Atf4*	AACCTCATGGGTTCTCCAGCGA	CTCCAACATCCAATCTGTCCCG
*Ddit3*	GGAGGTCCTGTCCTCAGATGAA	GCTCCTCTGTCAGCCAAGCTAG
*Hspa5*	TGTCTTCTCAGCATCAAGCAAGG	CCAACACTTCCTGGACAGGCTT

### RNA-seq analysis

Total RNA was isolated from dCLNs by TRIZOL™ reagent (Invitrogen, 15596018) according to the manufacturer’s instruction. Sample quality control was performed using RNA Nano 6000 Assay Kit of the Bioanalyzer 2100 system, and the Qubit Fluorometer (Thermo Fisher Scientific). All RNA sample processing (including linear RNA amplification and cDNA library generation) and RNA-seq were performed by Novogene Biotech, Inc. (Beijing, China). DESeq2 R package (1.20.0) was used to normalize the raw counts and perform differential expression analysis, with the P value corrected for multiple hypothesis testing using Benjamini–Hochberg false-discovery rate procedure (adjusted *P* value). Gene Ontology (GO) enrichment analysis of differentially expressed genes was implemented by the clusterProfiler R package with Fisher’s exact test, in which gene length bias was corrected. The minimum gene number per GO terms was set at ≥ 10. GO terms with corrected *P* value ≤ 0.05 considered significantly enriched by differential expressed genes.

### Enzyme-linked immunosorbent assay

The inflammatory cytokines (including IL-1β, IL-6 and TNF-α) in plasma or culture medium of BMDMs were examined by an enzyme-linked immunosorbent assay (ELISA) kit (Mouse-TNF-alpha ELISA Kit, KE10002; Mouse-IL-1-beta ELISA Kit, KE10003; Mouse-IL-6 ELISA Kit, KE10007, Proteintech) according to the manufacturer’s instructions.

### FACS sorting of macrophages in dCLN

To sort the macrophages in dCLN, the suspension of cells was achieved by extruding dCLNs through a 70-μm stainless steel-mesh cell strainer. Cell suspensions were then pelleted and resuspended in ice-cold FACS buffer containing anti-CD45−PE-Cy7 (1:100, clone 30-F11, Invitrogen) and anti-CD11b-FITC (1:100, clone M1/70, Invitrogen). Cells were then washed and resuspended in PBS buffer. Macrophages in dCLNs were then gated as CD45+ and CD11b+ and sorted into 15 mL centrifuge tubes using the FACS Aria II (BD Biosciences, Franklin Lakes NJ, USA).

### Isolation of bone-marrow-derived macrophages (BMDMs)

Murine BMDMs were isolated from bone marrow of C57BL/6 mouse femurs and tibias. The flushed, single-suspended, bone-marrow cells were treated with RBC Lysis buffer (eBioscience, 00-4300-54) and cultured in DMEM F12 50/50 media (Gibco) supplemented with l-glutamine, 10% fetal bovine serum, penicillin–streptomycin, and 20 ng/mL recombinant M-CSF (Peprotech). Culturing cells for 7 days in a humidified incubator with 5% CO_2_ can be stimulated by 100 nM α-syn monomer, 100 nM oligomer, or 400 nM α-syn monomer for 24 h for the following experiments. All BMDMs used in the study were confirmed to be over 90% CD11b^+^.

### Cell culture

Macrophage cell line RAW 264.7 (ATCC #TIB-71) were maintained in RPMI 1640 medium with 2 mM l-glutamine and 25 mM HEPES, supplemented with 10% fetal bovine serum. For Co-IP and immunofluorescence staining experiments, RAW 264.7 cells were cultured with 500 nM α-syn overnight.

### Co-IP

After incubating with 500 nM α-syn overnight, RAW 254.7 cell protein was extracted with tris buffer containing 1% NP-40 and protease inhibitor cocktail (Sigma). The lysate of cells was collected into a 1.5 mL tube and centrifuged at 14,000×*g* at 4 °C for 10 min. The supernatants of whole cell lysates were determined by BCA Protein Assay Kit. Then, 5 μg rabbit anti-GRP78 antibody (Abcam, ab21685), rabbit anti-human α-syn antibody (Abcam, ab138501) or rabbit control IgG (Sigma) was incubated with 500 μg protein at 4 °C overnight. Then, 50 μL Protein A/G Agarose (Santa Cruz) was added to the protein and antibody complex system and incubated at 4 °C for 2 h, which was collected by centrifugation at 14,000×*g*, 4 °C for 2 min, and washed three times with 0.5% NP-40 in tris buffer. The protein–antibody complex was then loaded onto SDS–PAGE. Rabbit anti-GRP78 (1:1000, Proteintech, 11587) and rabbit anti-human α-syn (1:4000, Abcam, ab138501) were used to detect the specific protein band on PVDF membrane.

### Flow cytometry analysis

Following i.c.m. injection of AF647-α-syn, dCLNs were harvested and extruded through a 70-μm stainless steel-mesh cell strainer. Cell suspensions of dCLNs were then pelleted, resuspended in ice-cold FACS buffer containing anti-CD45–PE-Cy7 (1:100, clone 30-F11, Invitrogen), anti-CD11b-FITC (1:100, clone M1/70, Invitrogen) and anti-F4/80-PE (1:100, clone BM8, Invitrogen). Macrophages containing α-syn in dCLNs were analyzed by BD FACS Calibur Flow Cytometer (BD Biosciences, Franklin Lakes NJ, USA). Flow cytometry data were processed by FlowJo software (FlowJo LLC, Ashland, OR, USA).

### Meso scale discovery multiplexed immunoassays

The detection and quantification for total and aggregated α-syn was based on a previously developed and validated assay [42]. Briefly, antibody against α-syn, MJFR-1 (ab138501, Abcam, only recognizes humanized α-syn) and antibody against conformation specific α-syn filaments, MJFR-14 (ab209538, Abcam, recognizes the structure of α-syn oligomers from both mouse and human species) were, respectively, biotinylated, linker conjugated, and coated onto standard 96-well U-Plex plates (Meso scale discovery). After washing three times with 150 μL Washing Buffer, 50 µL of diluted sample (diluted by Diluent 35 at a dilution ratio of 1:5) and calibrator (recombinant α-syn, Sino biological, and oligomeric α-syn, Proteos) were loaded to the immunoassay plate and incubated overnight at 4 °C on a shaker at 600 rpm. Next, the plate was washed three times using washing buffer followed by the addition of sulfo-TAG-labelled anti-α-syn antibody (BD42) and incubated at room temperature for 1 h on a shaker at 600 rpm. Finally, 150 μL 2× read buffer T was added into each well and plates were analyzed in a Quickplex SQ 120 (MSD, USA). Data analysis was performed with the MSD Discovery Workbench 3.0 Data Analysis Toolbox. Normalized concentration of total α-syn (pg/mg) was defined as total α-syn (ng/mL) divided by total protein (mg/mL), whereas normalized concentration of oligomeric α-syn (pg/ng) was defined as oligomeric α-syn (pg/mL) divided by normalized concentration of total α-syn (ng/mL).

### Pole test

Pole test was conducted as described previously with minor modifications [43]. Briefly, the mice were head-upward placed on the top of a vertically placed pole (40 cm in height and 1 cm in diameter). The time until all the legs of the mouse touched the floor was recorded. Time durations longer than 120 s or the mice falling from the pole were recorded as 120 s. All mice were trained two times per day for 3 days before the formal measurements.

### Rotarod test

Rotarod test was used to detect the ability of motor coordination on a rotating rod [44]. A 3 cm diameter rotating rod was started with a rotation speed of 5 rpm and accelerated at 0.1 rpm/s. The latency to fall from the rod in three consecutive trials was recorded, with a cutoff time of 2 min (*n* = 5 for each group). Prior to the test, all mice were trained for 3 times a day for 3 days to get accustomed to the apparatus.

### Quantification and statistical analysis

The relative fluorescent intensity, lymph node cross-sectional area, surface area of reticular fibers, medullary cord area and protein levels were measured by Image J (NIH). Statistical analyses were performed using Prism 9.0 (GraphPad, USA). The Shapiro–Wilk test and quantile–quantile plot were used to assess normal distribution of the data. For the data with normal distribution, the statistical significance was assessed by *t* test or one-way ANOVA analysis, followed with Bonferroni’s post-hoc test for multiple comparisons. For non-normal distribution data, the statistical significance was assessed by Mann–Whitney U test.

## Results

### Alterations in the structure and fluid dynamics in dCLN of A53T mice

**Fig. 1:**
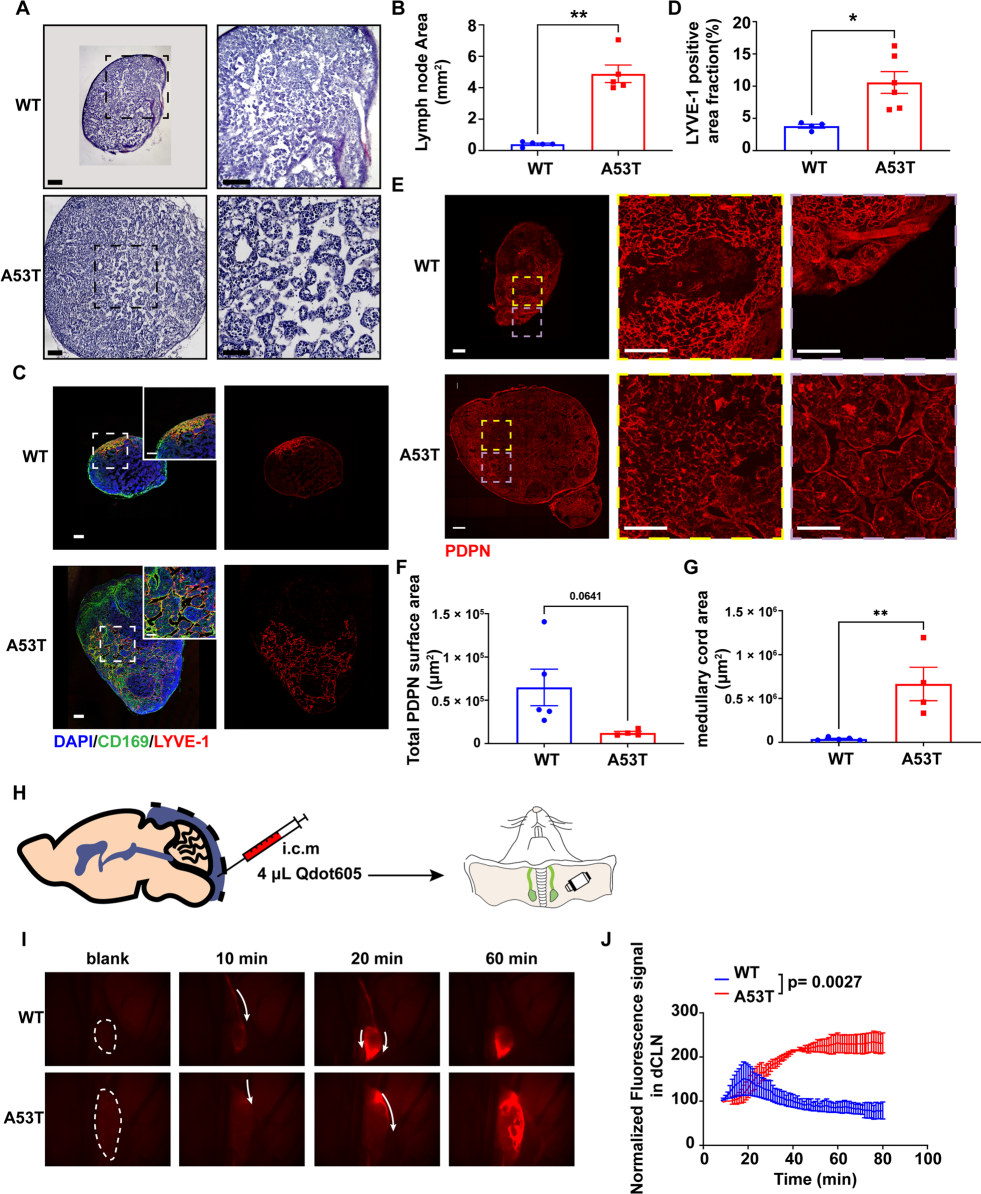
Tissue structural and fluid dynamics alteration in dCLNs of A53T mice. **A** Histology of representative dCLNs of WT and A53T mice. Scale bar, 200 μm. **B** Quantitative analysis of the cross-sectional area of dCLNs of WT and A53T mice. *N* = 5 independent animals in each group. **C** Representative fluorescence images of dCLNs of WT and A53T mice immunolabeled with LYVE-1 (red) and CD169 (green). Scale bar, 200 μm. **D** Quantitative analysis of the LYVE-1 positive area in dCLNs of WT and A53T mice. *N* = 4 independent animals in WT and *N* = 6 independent animals in A53T. **E** Representative fluorescence images of dCLNs of WT and A53T mice immunolabeled with PDPN. Yellow dotted box indicates reticular meshwork, and purple dotted box indicates medullary cord. Scale bar, 200 μm. **F** Quantitative analysis of the total surface area of PDPN positive reticular meshwork in dCLNs of WT and A53T mice. *N* = 5 independent animals in WT and *N* = 4 independent animals in A53T. **G** Quantitative analysis of the area of medullary cord in dCLNs of WT and A53T mice. *N* = 5 independent animals in WT and *N* = 4 independent animals in A53T. **H** Schematic of the experimental setup for the monitoring of lymph flow in dCLNs. **I** Representative images of dCLNs in WT and A53T mice from pre-infusion to 10 min, 20 min, 60 min post-infusion. White dotted circle indicates the dCLN. **J** Quantitative analysis of Qdot605 signal intensity over 80 min in dCLNs (relative to 8 min post-infusion). *t* Test was performed at signals between WT and A53T mice at 80 min. *N* = 4 independent animals. Values are means ± S.E.M, *t* test (**D**, **F**, **G**, and **J**), Mann–Whitney U test (**B**). *,* P* < 0.05; **, *P* < 0.01; ****, *P* < 0.0001

To evaluate the structural alterations in dCLN, we started with H&E stanning of the dCLN of WT and A53T mice. As demonstrated in Fig. 1A, B, compared with WT mice, lymphadenectasis was obviously detected in A53T mice, and the enlargement of the lymph sinus was highly significant. To directly detect the structural alterations in dCLN, a red fluorochrome Qdot605 was infused into the intra-cisterna magna of WT and A53T mice. Notably, Qdot605 was widespread in the whole dCLN of A53T mice, in clear contrast to WT dCLN, where the distribution of Qdot605 was relatively concentrated in the medulla (Additional file 1: Fig. S1). To further quantify the extent of the lymph sinus enlargement in dCLNs, the lymph sinus was immunolabelled with LYVE-1. As shown in Fig. 1C, D, the areas outlined by LYVE-1 in A53T mice were significantly larger than those of the WT group.

Lymph node is typically supported by the reticular fibers composed of fibroblastic reticular cells expressing podoplanin (PDPN) [40]. To further assess the structural alteration in the dCLN of A53T mice, the reticular meshwork in dCLN was immunolabeled with PDPN. As shown in Fig. 1E, the reticular meshwork was mainly distributed in the paracortex and medulla of dCLN. The quantification data of the surface area of reticular fibers (Fig. 1F) indicated that the reticular meshwork in the dCLN of A53T mice was relatively looser than that of WT group. Along with the enlarged lymph sinus, the medullary cords in the dCLN of A53T mice were also significantly larger than those of the WT mice (Fig. 1G).

Structural alterations in a lymph node are likely to alter lymphatic fluid drainage. To probe this possibility, Qdot 605 fluorescence signal was infused into the intra-cisterna magna (detailed scheme in Fig. 1H) and recorded with multiphoton microscopy every 30 s to analyze the dynamic flow in the dCLN. In WT mice, Qdot 605 was drained from CSF into the dCLN within 20 min after the injection and then quickly concentrated in the medullary sinus (Fig. 1I, J). In A53T mice, Qdot605 remained in the subcapsular sinus for a much longer time (WT: 15–25 min; A53T: 45–60 min) before moving into the medullary sinus slowly. In addition, in the dCLN of A53T mice, a part of Qdot605 was transported across the subcapsular sinus towards paracortex, much beyond the medullary sinus (Additional file 3: Video S1, Additional file 4: Video S2).

### Increased inflammation and dysfunctional macrophages in dCLN of A53T mice

**Fig. 2:**
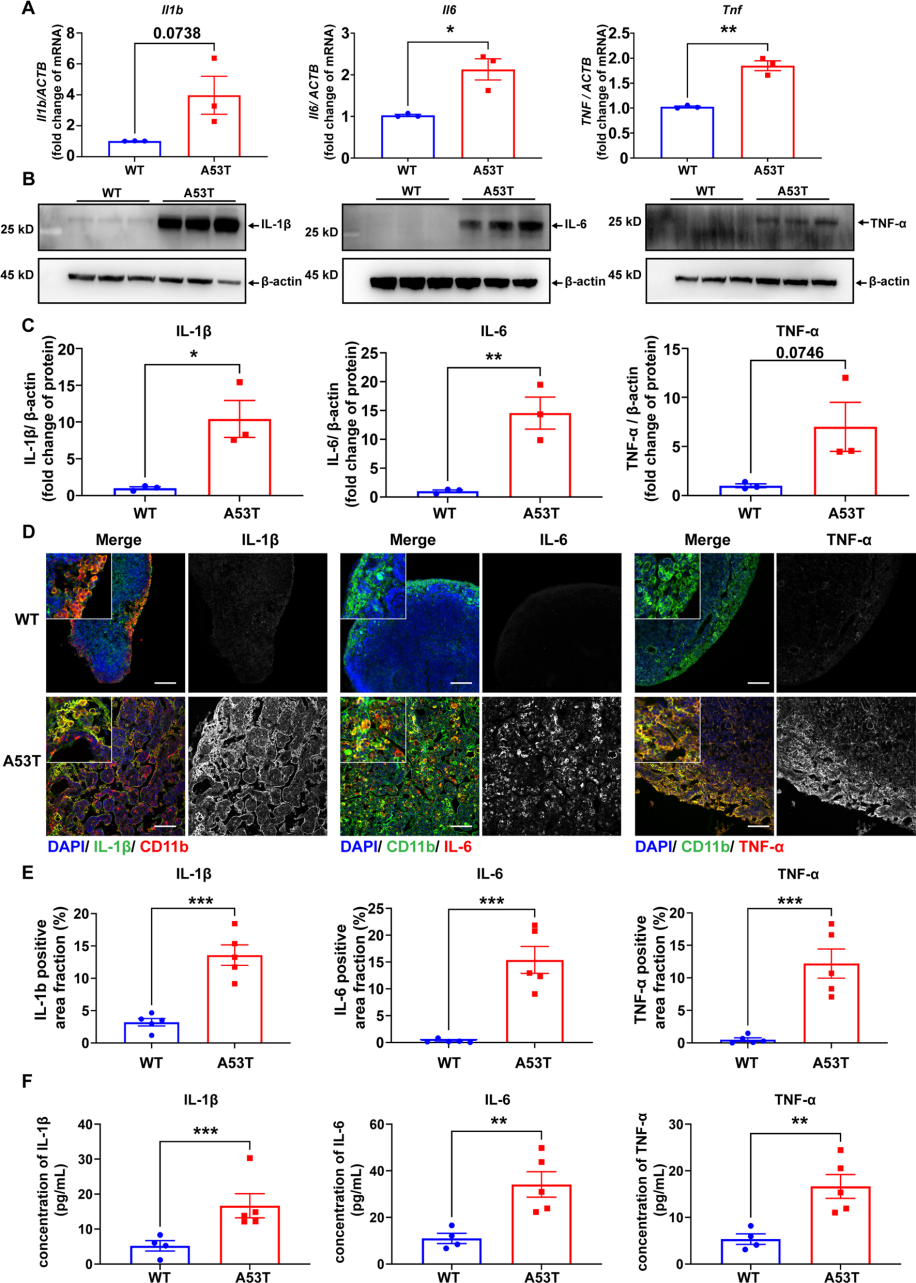
Peripheral inflammation in A53T mice. **A** Quantitative analysis of mRNA levels of *Il1b*, *Il6* and *Tnf* in dCLNs of WT and A53T mice using qPCR. *N* = 3 independent animals in each group. **B** Western blot to assess the level of IL-1β, IL-6 and TNF-α in dCLNs of WT and A53T mice. **C** Quantitative analysis of the protein level of IL-1β, IL6 and TNF-α in dCLNs of WT and A53T mice, *N* = 3 independent in each group (3 samples were pooled from 6 animals). **D** Representative fluorescence images of dCLNs from WT and A53T mice immunolabeled by CD11b (red or green) with IL-1β (green), IL-6 (red), or TNF-α (red). Scale bar, 100 μm. **E** Quantitative analysis of IL-1β, IL6 and TNF-α positive area in dCLNs of WT and A53T mice. *N* = 5 independent animals in WT and A53T. **F** Quantitative analysis of the levels of IL-1β, IL6 and TNF-α using ELISA, in plasma of WT and A53T mice. *N* = 4 independent animals in WT group and *N* = 5 independent animals in A53T group. Values are means ± S.E.M, *t* test. *, *P* < 0.05; **, *P* < 0.01; ***, *P* < 0.001

The increased size of lymph node objectively reflected its response to peripheral inflammation, which is one of the most important functions of lymph node. To explore the extent of inflammation in the dCLN of A53T mice, a set of inflammatory cytokines, including IL-1β, IL-6 and TNF-α were measured in the dCLN. As shown in Fig. 2A–C, the mRNA and protein levels of IL-1β, IL-6 and TNF-α were significantly increased in the dCLN of A53T mice as compared with WT mice. Besides, the significantly increased immunofluorescence signals of IL-1β, IL-6 and TNF-α in the dCLN of A53T mice are mainly concentrated in the medulla of lymph node and significantly co-localized with CD11b and F4/80 signals, which labelled macrophages (Fig. 2D, E and Additional file 1: Fig. S2). In addition, the levels of IL-1β, IL-6, and TNF-α in plasma were also significantly increased in A53T mice (Fig. 2F).

### Activation of macrophages by lymphatic drained oligomeric α-syn

**Fig. 3:**
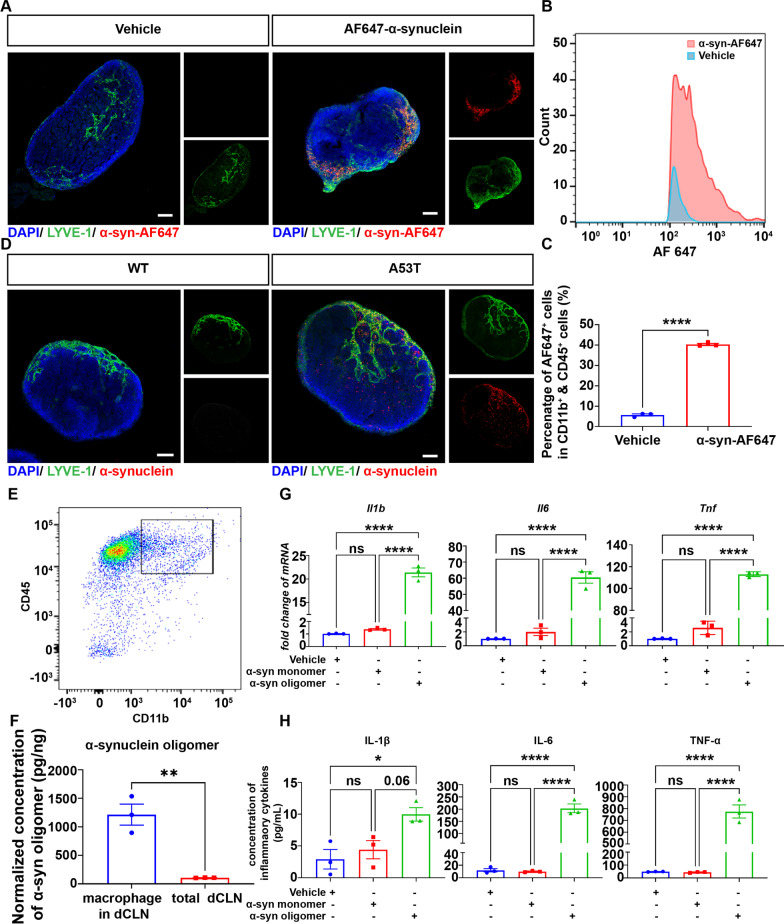
α-Synuclein in CNS was drained to the macrophage and activated it in the dCLN through meningeal lymphatics. **A** Representative fluorescence images of dCLNs of C57 mice injected (i.c.m) with vehicle or AF647-α-synuclein (red). Scale bar, 200 μm. **B** Number of macrophages containing AF647-α-synuclein in dCLNs of C57 mice injected (i.c.m) with vehicle or AF647-α-synuclein was evaluated by flow cytometry. The macrophages were labelled by CD11b-FITC and CD45-PE-Cy7. **C** Quantitative analysis of the number of macrophages containing AF647-α-synuclein in dCLNs of C57 mice. **D** Representative fluorescence images of dCLNs in WT and A53T mice. α-Synuclein in dCLN was immunolabelled by MJFR-1 (red). Scale bar, 200 μm. **E** Macrophages in dCLN were sorted by FACS. The macrophages were labelled by CD11b-FITC and CD45-PE-Cy7. **F** Quantitative analysis of α-synuclein oligomer in macrophages of dCLNs and total dCLNs using MSD. *N* = 3 independent experiments in each group. **G** Quantitative analysis mRNA levels of *Il1b*, *Il6* and *Tnf* in BMDMs treated with vehicle control, 100 nM α-syn monomer, or 100 nM α-syn oligomer using qPCR. *N* = 3 independent experiments in each group. **H** Quantitative analysis of the levels of IL-1β, IL6 and TNF-α using ELISA, released by BMDMs treated with vehicle control, α-syn monomer, or α-syn oligomer. *N* = 3 independent sample pools in each group. Values are means ± S.E.M, *t* test (**C**, **F**), one-way ANOVA test (**G**, **H**). *, *P* < 0.05; **, *P* < 0.01; ****, *P* < 0.0001

Given obviously higher levels of inflammatory cytokines in the macrophages of A53T mouse dCLN (Fig. 2) and our previous finding that oligomeric α-syn has an immune-regulation effect on CNS microglia [45], we hypothesized that oligomeric α-syn drained from the CNS through meningeal lymphatics elicited the inflammatory activation of macrophages and contributed to the peripheral inflammations in the dCLN. To test the hypothesis, we first asked whether α-syn from the CNS can enter the macrophage of the dCLN. To trace the lymphatic drainage route of α-syn from the CNS, Alexa Flour 647-labelled α-syn (α-syn-AF647) was intra-cisternally injected into the CSF circulation of C57Bl/6J mice (Additional file 1: Fig. S3A). The fluorescence signal of α-syn around meningeal lymphatic vessels and in the dCLNs indicated that α-syn from the CNS could be drained to the dCLNs through the meningeal lymphatics (Fig. 3A, Additional file 1: Fig. S3B–G). In addition, to avoid potential interference of fluorescence labelling, we also intra-cisternally injected α-syn without fluorescence label into the CSF circulation of C57Bl/6J mice. WB of the dCLNs further indicated that α-syn from the CNS was readily drained to the dCLNs (Additional file 1: Fig. S3H, I). Moreover, the number of macrophages containing AF647 fluorescence signal in the dCLN of the α-syn-AF647 group was significantly more than that of the vehicle-treated group as determined by flow cytometry (Fig. 3B, C). To exclude the possible interference of the procedure of exogenous injection, we directly detected α-syn in the macrophages of dCLN in A53T mice. The immunofluorescence of dCLN revealed a significant lymphatic drainage of α-syn from the CNS to the dCLNs of A53T mice as observed in Fig. 3D. To directly determine the accumulation of α-syn in the macrophages of dCLNs in A53T mice, fluorescence-activated cell sorting (FACS) was applied to sort the macrophages. The level of α-syn in macrophages was then determined with MSD assay (Fig. 3E, F), which detected both total and oligomeric α-syn in the macrophages of dCLN in A53T mice (Additional file 1: Fig. S4). Remarkably, the level of oligomeric α-syn in the macrophages was significantly higher than that in the lysate of total dCLNs (Fig. 3F).

To determine whether oligomeric α-syn provoked the inflammatory activation of the macrophages in the dCLN of A53T mice, bone-marrow-derived macrophages (BMDMs) were isolated and used in a set of in vitro experiments. The mRNA and protein levels of inflammatory cytokines, including IL-1β, IL-6 and TNF-α, in the macrophages, stimulated by vehicle control, α-syn monomers or oligomers, were measured by qPCR and ELISA, respectively. The levels of inflammatory cytokines in the macrophages stimulated by α-syn oligomers were significantly higher than those induced by the vehicle control or α-syn monomers, suggesting that oligomeric α-syn is indeed essential to induce the inflammatory activation of macrophages (Fig. 3G, H).

### Endoplasmic reticulum stress in the dCLN of A53T mice

**Fig. 4:**
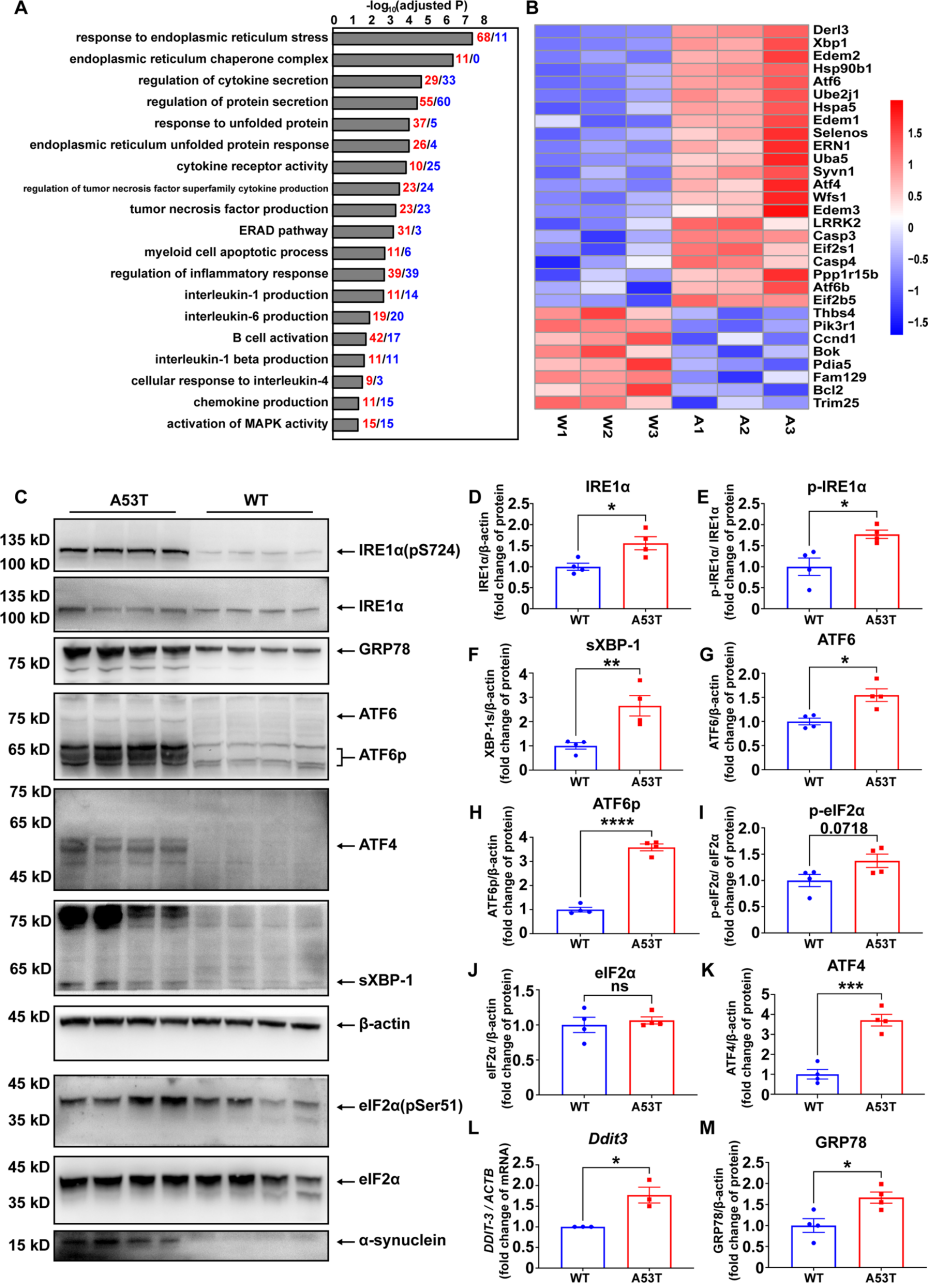
Endoplasmic reticulum stress was increased in the dCLN of A53T mice. **A** Functional enrichment of differentially expressed genes in dCLNs of WT and A53T mice. Red represented the number of increased genes and blue represented the number of decreased genes. **B** Heat map partly showing the relative expression levels of genes involved in response to endoplasmic reticulum stress signaling pathway. Red represents gene expression levels above the mean; blue represents gene expression levels below the mean. *N* = 3 independent in each group (3 WT samples were pooled from 6 animals; 3 A53T samples were pooled from 4 animals). **C** Western blot to assess the level of p-IRE1α, IRE1α, GRP78, ATF6, ATF6-p, ATF4, sXBP-1, p-eIF2α, eIF2α and α-synuclein in dCLNs of WT and A53T mice. **D–K** Quantitative analysis of the level of IRE1α, p-IRE1α, sXBP-1, ATF6, ATF6-p, p-eIF2α, eIF2α and ATF4 in dCLNs of WT and A53T mice, *N* = 4 independent in WT group (4 WT samples were pooled from 8 animals) and *N* = 4 independent animals in A53T group. **L** Quantitative analysis of *Ddit3* mRNA levels using qPCR, in dCLNs of WT and A53T mice. *N* = 3 independent animals in each group. **M** Quantitative analysis of the level of GRP78 in dCLNs of WT and A53T mice. Values are means ± S.E.M, *t* test. *, *P* < 0.05; **, *P* < 0.01; ***, *P* < 0.001; ****, *P* < 0.0001

To explore the mechanism underlying the observed peripheral inflammation and lymph node enlargement in A53T mice, RNA-seq was performed on the dCLN of WT and A53T mice. Differential expression analysis showed that among 27,379 detected genes, 1919 genes were up-regulated and 1643 genes down-regulated in the dCLN of A53T mice (Additional file 1: Fig. S5A and Additional file 2: Table S2). Principal component analysis (PCA) showed two distinct disparate clusters between WT and A53T mice (Additional file 1: Fig. S5B). Gene Ontology (GO) enrichment analysis revealed alterations of multiple pathways related to endoplasmic reticulum stress (ER stress), with the cytokine production and secretion (including interleukin-1, interleukin-6, and tumor necrosis factor) pathway up-regulated as well (Fig. 4A and Additional file 1: Fig. S5C). The heatmap of the relative expression levels of genes related to unfolded protein response (UPR), ER-associated protein degradation (ERAD), and apoptosis are all indicative of an increased ER stress induced in the dCLN of A53T mice (Fig. 4B and Additional file 1: Fig. S6). Notably, the mRNA levels of the typical genes directly involved in the response to ER stress, including *Ern1*, *Xbp-1*, *Atf6*, *Atf4*, *Ddit3*, and *Hspa5*, were further determined to be significantly increased in the dCLNs of A53T mice (Additional file 1: Fig. S5D).

To confirm the increased ER stress, the UPR pathways, including the IRE1-α signaling axis, the ATF6α route and the PERK-signaling axis [46], were analyzed at protein levels by Western blot (Fig. 4C). IRE1α protein in the ER membrane significantly increased (Fig. 4D), along with a higher phosphorylation level of IRE1α in A53T mice (Fig. 4E). The transcription factor, spliced XBP-1 (sXBP-1), the product dependent on the phosphorylation of IRE1α, was also significantly increased in the dCLN of A53T mice (Fig. 4F). In ATF6α pathway, both the full-length ATF6α and the transcription factor, processed ATF6α (ATF6p), generated by Golgi apparatus’ intramembrane proteolysis, significantly increased in A53T mice, compared with WT counterparts (Fig. 4G, H). In PERK-signaling axis, activation of PERK can directly phosphorylate the translation initiation factor eIF2α, which subsequently facilitates the production of transcription factors (ATF4 and Ddit3) [47]. In the dCLN of A53T mice, the phosphorylation of eIF2α, protein level of ATF4 and mRNA level of *Ddit3* were all significantly higher than those in the WT mice (Fig. 4I, K, L). Moreover, as the key factor initiating the UPR, GRP78, the ER chaperone, significantly increased in A53T mice (Fig. 4M). Altogether, the significantly increased UPR-related proteins indicate profound ER stress occurred in the dCLN of A53T mice.

**Fig. 5:**
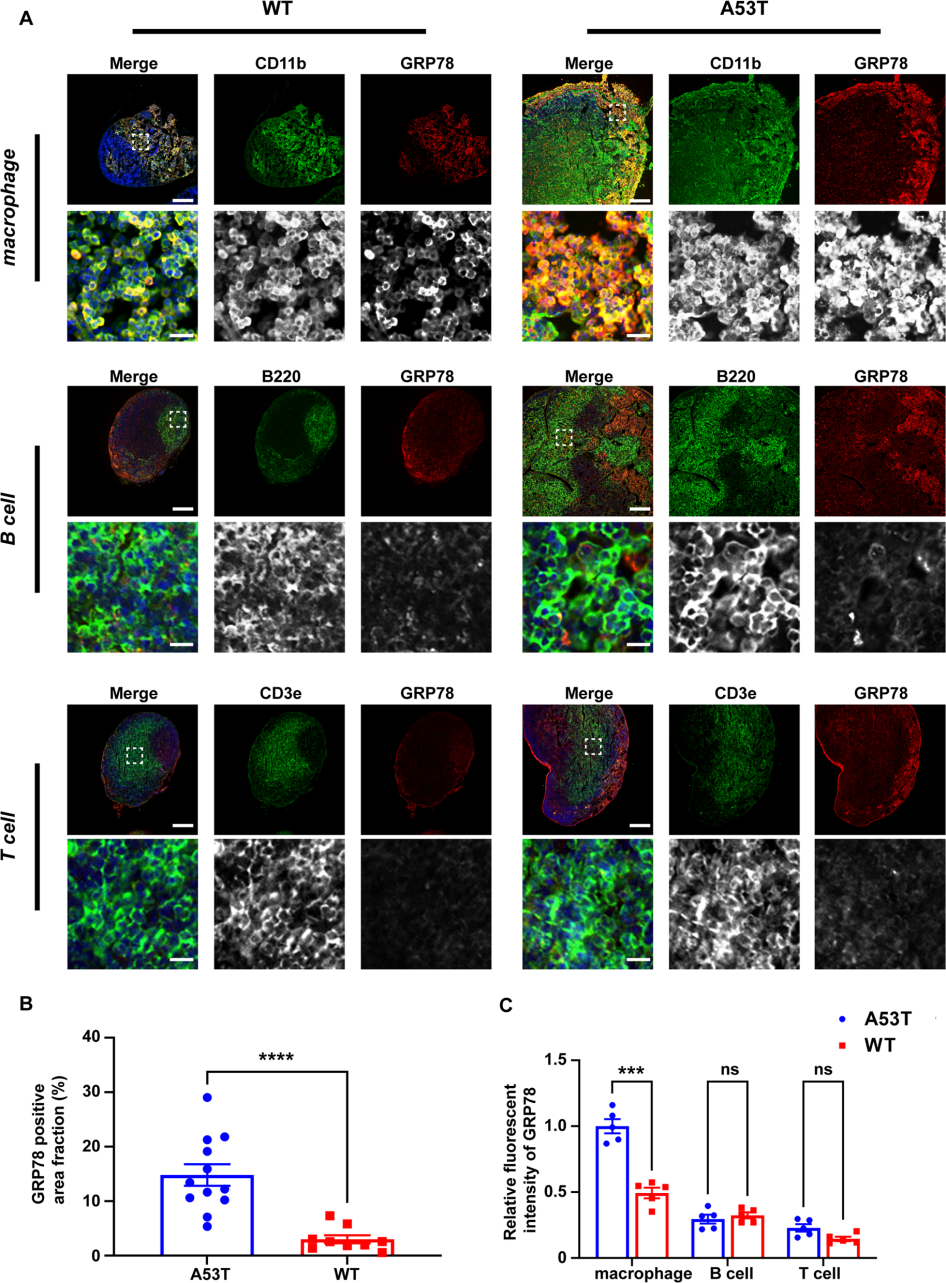
Endoplasmic reticulum stress in dCLNs of A53T mice mainly existed in macrophages. **A** Representative fluorescence images of GRP78 in macrophages (CD11b positive), B cells (B220 positive) and T cells (CD3e positive) in dCLNs of WT and A53T mice. Scale bar, 200 μm in upper images, 20 μm in lower images. **B** Quantitative analysis of the intensity of GRP78 in dCLNs of WT and A53T mice. *N* = 9 images from 3 independent animals in WT group and *N* = 12 images from 4 independent animals in A53T group. **C** Quantitative analysis of the intensity of GRP78 in macrophages, B cells and T cells in dCLNs of WT and A53T mice. *N* = 5 independent animals. Values are means ± S.E.M, *t* test (**B**), two-way ANOVA test (**C**). ns, not significant; ***, *P* < 0.001; ****, *P* < 0.0001

To determine whether the increased ER stress happened specifically in the macrophages of the dCLN in A53T mice, the levels of ER stress in the macrophages, T-cells and B-cells were determined by immunofluorescence, respectively. Considering the key role of GRP78 in ER stress and the increased protein level of the GRP78 in A53T mice, we selected GRP78 as the indicator of the ER stress. CD11b (or F4/80), B220, and CD3e were used to indicate macrophages, B- and T-cells, respectively. The fluorescence signal of GRP78 in A53T mice was significantly higher than that of WT mice (Fig. 5A, B and Additional file 1: Fig. S2C), consistent with the Western blot results shown in Fig. 4M. More importantly, in the dCLN of A53T mice, the signal of GRP78 in CD11b positive cells or F4/80 positive cells was significantly higher than that in B220 or CD3e positive cells (Fig. 5A, C and Additional file 1: Fig. S2C), indicating that macrophages are the main immune cell types involved in the ER stress.

### α-Syn is involved in the endoplasmic reticulum stress

**Fig. 6:**
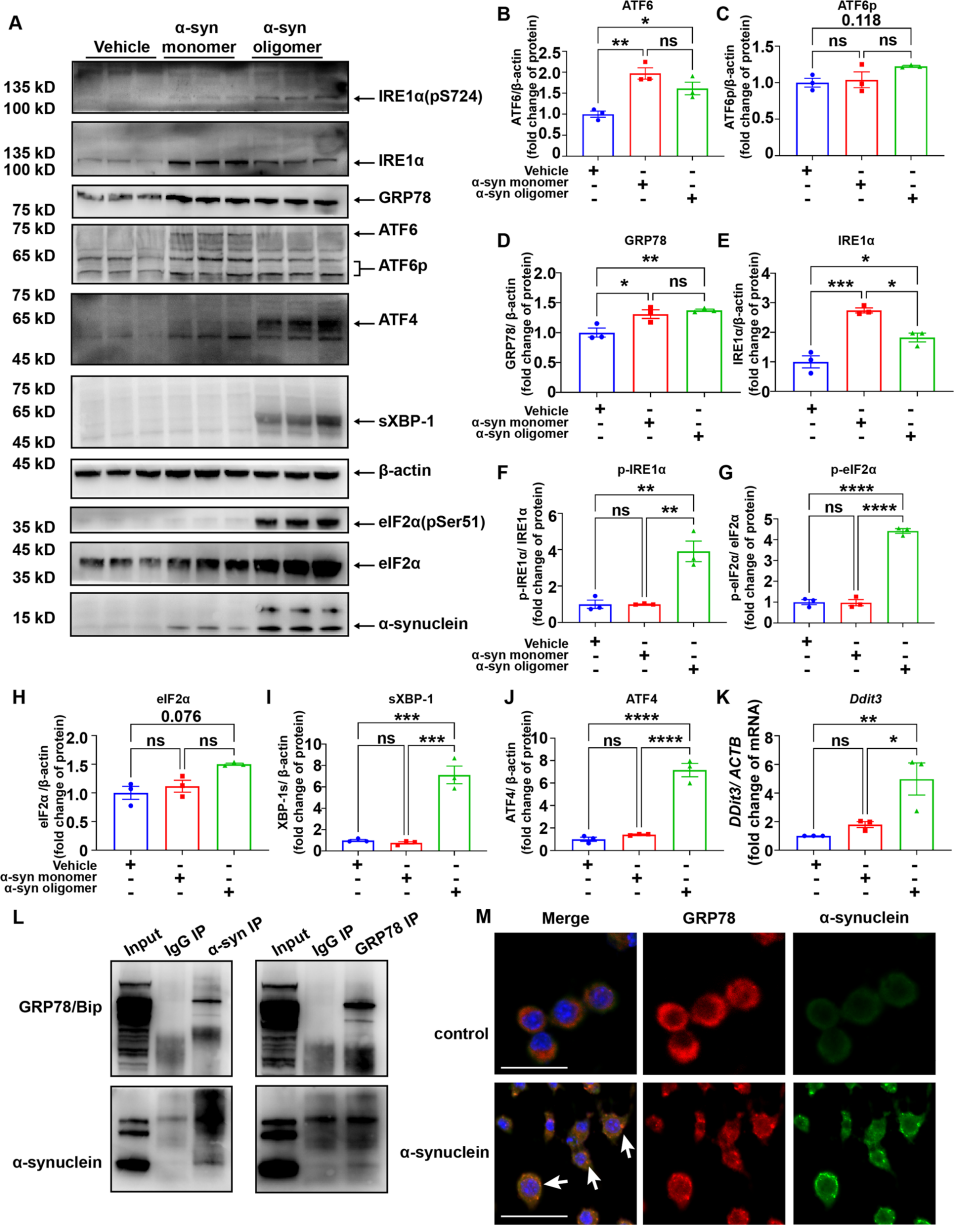
α-Synuclein aggregate elicited severe ER stress in the macrophage. **A** Western blot to assess the levels of p-IRE1α, IRE1α, GRP78, ATF6, ATF6-p, ATF4, sXBP-1, p-eIF2α, eIF2α and α-synuclein in BMDMs treated with vehicle control, 100 nM α-synuclein monomer or 100 nM α-synuclein oligomer. **B**–**J** Quantitative analysis of the level of ATF6, ATF6-p, GRP78, IRE1α, p-IRE1α, p-eIF2α, eIF2α, sXBP-1, and ATF4 in BMDMs treated with vehicle control, α-synuclein monomer or α-synuclein oligomer. *N* = 3 independent experiments in each group. **K** Quantitative analysis of *Ddit3* mRNA levels in BMDMs treated with vehicle control, α-synuclein monomer or α-synuclein oligomer using qPCR. *N* = 3 independent experiments in each group. **L** Interaction between α-syn and GRP78 was examined by co-IP in RAW 264.7 cells. **M** Co-localization of α-syn (green) and GRP78 (red) in RAW 264.7 cells after treatment with α-syn. Scale bar, 50 μm. Values are means ± S.E.M, one-way ANOVA test. ns, not significant; *, *P* < 0.05; **, *P* < 0.01; ***, *P* < 0.001; ****, *P* < 0.0001

To explore the possibility of a direct involvement of α-syn in the ER stress of the macrophage in the dCLN of A53T mice, BMDMs were stimulated by α-syn monomers or oligomers. As observed in Fig. 6A, although the protein levels of ATF6α, GRP78, and IRE1α were significantly increased in the macrophages stimulated by both oligomeric and monomeric α-syn (Fig. 6B–E), compared with α-syn monomers, the phosphorylation levels of IRE1α and eIF2α (Fig. 6F–H), the protein levels of sXBP-1 and ATF4, and the mRNA level of *Ddit3* (Fig. 6I–K) in the macrophages were only significantly increased when stimulated by oligomeric α-syn, indicating that it was the oligomeric α-syn that preferentially elicited a potent ER stress in the macrophage. It is worth noting that, more α-syn remained in the macrophage stimulated by oligomers than monomers with an equivalent loading amount of the protein (Fig. 6A). Besides, even a high level of α-syn monomers failed to induce the increase of ER stress-related proteins in the macrophages (Additional file 1: Fig. S7).

The UPR is known to be dependent on the binding between misfolded proteins and GRP78 [48]. To probe the mechanism of ER stress elicited by α-syn, we took advantage of co-IP and immunofluorescent staining, revealing a direct interaction between GRP78 and α-syn (Fig. 6L, M). To directly link the ER stress in the dCLN of PD with the lymphatic drainage of α-syn from the CNS, we intracisternally injected oligomeric α-syn into the CSF circulation of C57 mice and collected the dCLNs of C57 mice 24 h later, using Western blot to evaluate the intensity of ER stress-related proteins in the dCLN. Compared with C57 mice injected with vehicle control, the phosphorylation levels of IRE1α and eIF2α as well as the protein levels of ATF6p and GRP78 were increased in the dCLN of C57 mice injected with oligomeric α-syn (Additional file 1: Fig. S8). These findings suggest that the lymphatic drainage of oligomeric α-syn does contribute to the ER stress in the dCLNs.

### Endoplasmic reticulum stress mediates macrophage activation and peripheral inflammation

**Fig. 7:**
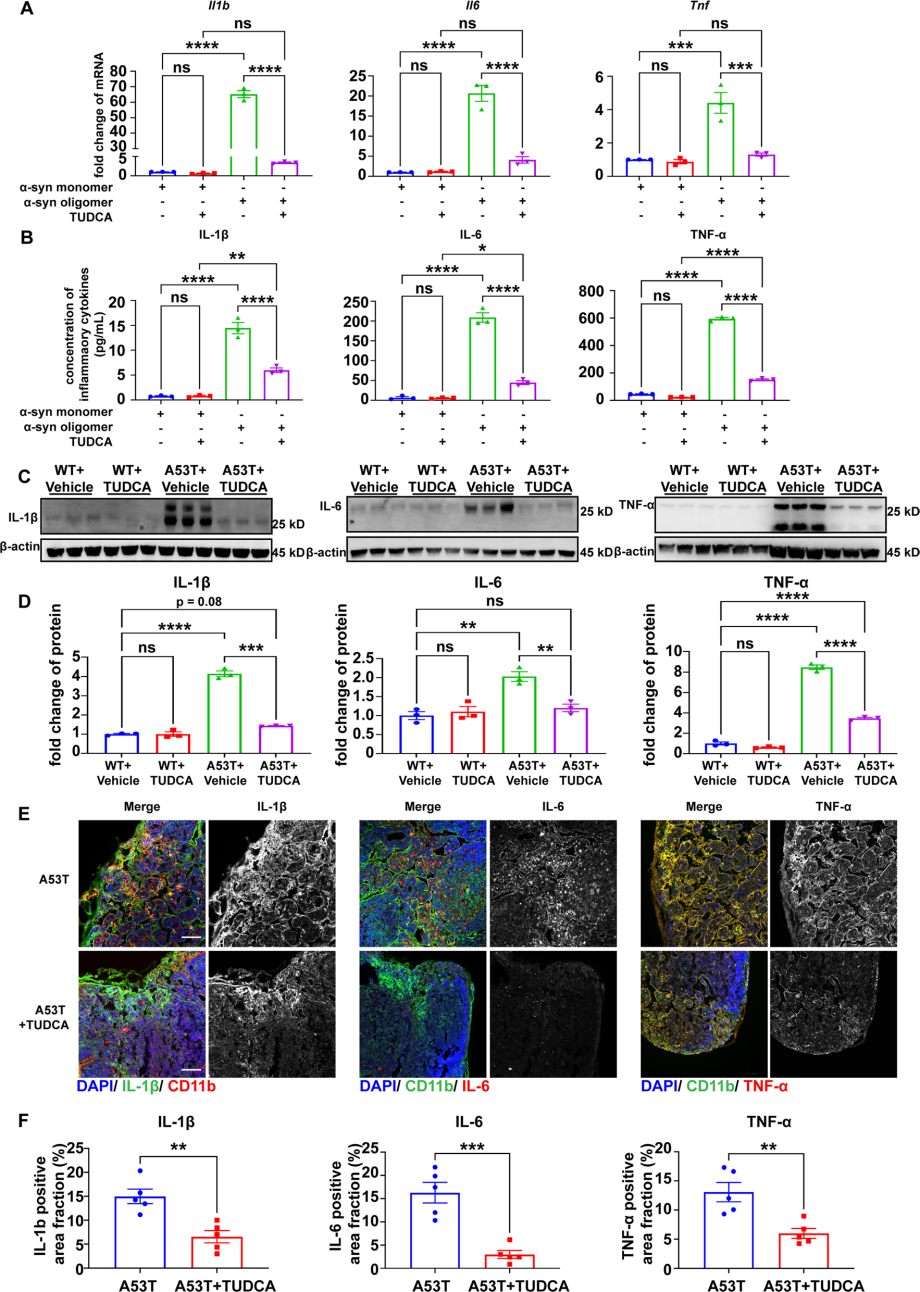
ER stress was involved in the peripheral inflammation in PD mice. **A** Quantitative analysis mRNA levels of *Il1b*, *Il6* and *Tnf* in BMDMs treated with 100 nM α-syn monomer or oligomer and vehicle control or 100 μM TUDCA using qPCR. *N* = 3 independent experiments in each group. **B** Quantitative analysis of the levels of IL-1β, IL6 and TNF-α released by BMDMs treated with 100 nM α-syn monomer or oligomer and vehicle control or 100 μM TUDCA using ELISA. *N* = 3 independent in α-syn oligomer group, *N* = 6 independent in α-syn oligomer and TUDCA group. **C** Western blot to assess the level of IL-1β, IL6 and TNF-α in dCLNs of WT or A53T mice treated with vehicle control and TUDCA (100 mg/kg). **D** Quantitative analysis of the levels of IL-1β, IL6 and TNF-α in dCLNs of WT or A53T mice treated with vehicle control and TUDCA. *N* = 3 independent in each group (3 WT + Vehicle samples were pooled from 7 animals; 3 WT + TUDCA samples were pooled from 7 animals; 3 A53T + Vehicle samples were pooled from 5 animals; 3 A53T + TUDCA samples were pooled from 6 animals). **E** Representative fluorescence images of IL-1β, IL6 and TNF-α in dCLNs of A53T mice treated with or without TUDCA. Scale bar, 100 μm. **F** Quantitative analysis of IL-1β, IL6 and TNF-α positive area in dCLNs of A53T mice treated with or without TUDCA. *N* = 5 independent animals in each group. Values are means ± S.E.M, one-way ANOVA test (**A**, **B**, **D**), *t* test (**F**). *, *P* < 0.05; **, *P* < 0.01; ***, *P* < 0.001; ****, *P* < 0.0001

To further investigate the relationship between the ER stress and the macrophage activation by oligomeric α-syn, the ER stress inhibitor, TUDCA [49] was applied to alleviate the ER stress in macrophages with α-syn stimulation. The results showed that the treatment of TUDCA significantly reduced the increased inflammatory cytokines in the macrophages stimulated by oligomeric α-syn, with no apparent changes observed in the macrophages stimulated by monomeric α-syn (Fig. 7A, B), indicating that α-syn oligomers contribute to the inflammatory activation of macrophages at least in part via ER stress. To further confirm this phenomenon in vivo, TUDCA was applied on the A53T mice, and the expression levels of IL-1β, IL-6 and TNF-α were determined by Western blot and immunofluorescence. As observed in Fig. 7C–F, compared with vehicle control, TUDCA treatment significantly decreased the levels of IL-1β, IL-6 and TNF-α in the dCLN of A53T mice, but not in that of the WT mice (Fig. 7C, D).

To determine the potential of TUDCA to prevent PD-related symptoms, motor function assessments, using the pole test and rotarod test, were conducted in WT and A53T mice treated with vehicle or TUDCA. The A53T mice treated with TUDCA tented to hold more time on the rod and spend less time to descend the pole than vehicle control (Additional file 1: Fig. S9A, B), although no statistical significance was achieved. Similarly, the number of TH positive dopaminergic neurons in the substantia nigra of A53T mice was rescued slightly, although not statistically significant, by TUDCA treatment (Additional file 1: Fig. S9C, D).

## Discussion

The major findings of this study include: (1) the CNS oligomeric α-syn potently activated the macrophages in dCLN through the meningeal lymphatics, resulting in alterations in the structure and fluid dynamics of the dCLN of A53T mice; and (2) the ER stress was involved in the activation of macrophages induced by oligomeric α-syn and likely contributed to the peripheral inflammation in A53T mice. This newly identified process may shed light on the mechanisms triggering peripheral inflammation in PD patients.

Lymph nodes are secondary lymphoid tissues, the key components for immune response, where normal and potentially harmful materials in the lymph fluid from the upstream were disposed or neutralized [38]. After reaching the subcapsular sinus (SCS) lumen through afferent lymphatic vessels, lymph fluid usually enters the conduit system (a meshwork of reticular collagen fibrils reaching deep into the paracortex) or gets delivered directly into the medulla sinus [50]. In this investigation, the alterations in microanatomical structure in the dCLN of A53T mice mainly occurred in the medulla and paracortex, manifesting as enlarged medullary sinus and loose reticular meshwork (Fig. 1C, E). The enlarged medullary sinus retained more lymphatic fluid, while loose reticular meshwork likely contributed to slower transport velocity in the conduit system [51], both of which could lead to a slower lymph flow in the dCLN of A53T mice (Fig. 1I, J, Additional file 3: Video S1, Additional file 4: Video S2).

In general, the size of lymph node increases to meet the demands of immune response to certain toxic contents in lymph fluid [52]. Increased inflammatory cytokines were obvious in the dCLN of A53T mice, especially in the macrophages of the medulla area (Fig. 2A–E). In addition, increased inflammatory responses were also observed in the plasma of A53T mice (Fig. 2F). Of note, peripheral inflammation has been widely reported in PD patients [20, 53], although the mechanisms involved remain to be investigated further. α-Syn produced in the line of A53T mice used in the current investigation is under a prion promotor, i.e., peripheral inflammation has to be a secondary effect of accumulation of misfolding α-syn aggregate in the CNS. Transportation of CNS α-syn to the blood has been demonstrated by many groups [54, 55], and as indicated earlier, this process is likely to also involve the lymphatic system and BBB.

In line with our previous observations, i.e., oligomeric α-syn activates microglia with the production of pro-inflammatory mediators [45], we observed α-syn drained from CSF potently activated the macrophages of dCLN (Fig. 2) in this study. Our results indicated that macrophage is the main immune cell type involved in response to oligomeric α-syn in dCLN as well as in the peripheral circulatory system [22, 23]. In PD patients, however, it would be rather challenging to differentiate the effects of lymphatic draining α-syn vs those transported via BBB directly in terms of activating peripheral immune cells.

To explore the mechanism underlying the lymph node enlargement and related inflammation in A53T mice, we performed RNA-seq on the dCLN of WT and A53T mice and revealed alterations of multiple pathways related to ER stress (Fig. 4). ER stress is typically attributed to protein misfolding or accumulation of misfolded proteins, which further triggers the unfolded protein response (UPR) [47]. UPR, composed of a collection of signaling pathways to manage ER stress, is likely a response and an important indicator to an increased ER stress. In an overt ER stress, UPR would be one of the key processes leading to inflammation [47, 56–58]. In this study, we detected significantly upregulated UPR both in the macrophages of dCLN of A53T mice (Fig. 5) and BMDMs stimulated by oligomeric α-syn (Fig. 6), consistent with the report that the aggregation of misfolded α-syn in the CNS could elicit ER stress in neurons [59]. Thus, we speculated that oligomeric α-syn drained from the CNS could activate macrophages in the dCLN of A53T mice via eliciting ER stress in macrophages, contributing to lymph node enlargements and increased peripheral inflammation. For this purpose, TUDCA, a chemical chaperone which stabilizes the structure and facilitates the folding of mis-folded aggregated proteins to suppress ER stress [60–62], was applied to BMDMs in vitro as well as in A53T mice (Fig. 7). Quite remarkably, TUDCA not only suppressed the inflammation of BMDMs stimulated by oligomeric α-syn but also directly in the dCLN of A53T mice.

How might oligomeric α-syn mediate ER stress? The initiation of UPR is heavily dependent on the binding between chaperone GRP78 and misfolded proteins [48], oligomeric α-syn in the context of our study. Given the interaction between α-syn and GRP78 (Fig. 7), it is reasonable to hypothesize that the accumulated oligomeric rather than monomeric α-syn interacts with GRP78 inducing ER stress in the macrophages, whether in vitro or in vivo. To cope with increased ER stress, the activation of UPR could increase the protein degradative pathways including ER‑associated degradation (ERAD) and autophagy [47, 48]. Notably, the level of remaining α-syn in the BMDMs treated with oligomeric α-syn was higher than that in the BMDMs treated with equivalent monomeric α-syn (Fig. 6A), suggesting that the protein degradation pathways activated by UPR were insufficient to cope with the oligomeric α-syn, which further aggravated the accumulation of misfolded α-syn oligomers and more severe ER stress in BMDMs, leading to the inflammatory activation of macrophage. The specificity of the effect induced by oligomeric α-syn is unknown, since we did not investigate other misfolded proteins, such as Aβ and tau protein, which are also drained by meningeal lymphatics in other diseases, including AD [35, 36].

To further evaluate the contribution of lymphatic drainage of α-syn from the CNS to the ER stress in the macrophages of the dCLN in A53T mice, we intracisternally injected oligomeric α-syn into the CSF circulation of C57 as well as A53T mice. Although direct injection of oligomeric α-syn into CSF circulation of C57 mice cannot elicit the equally serious ER stress in dCLN as A53T mice, our results demonstrated a weak but unequivocal increase of ER stress in dCLN of C57 mice induced by α-syn oligomers (Additional file 1: Fig. S6).

Recently, peripheral inflammatory mechanisms have been investigated and considered as a potential therapeutic target for PD [63, 64]. In this study, however, although TUDCA treatment clearly alleviated peripheral inflammation, the rescue of the motor function (Additional file 1: Fig. S9A, B) and neuronal damage in A53T mice (Additional file 1: Fig. S9C, D) was not statistically significant. In future studies, more investigations, including using a different treating regimen, is needed to fully explore the neuroprotective effect of TUDCA and related drugs.

## Conclusions

Taken together, several lines of evidence collected in our study suggest that lymph node enlargement is associated with macrophage activation and peripheral inflammation in PD mice, and that one of the key pathogeneses relates to the ER stress induced by oligomeric α-syn. Further investigations should be conducted to explore the possibility of modulating peripheral inflammation or even treating PD via regulating ER stress.

## Supplementary Information

**Additional file 1: Figure S1.** Distribution of Qdot605 in dCLNs of A53T mice. **A** Representative fluorescence images of dCLNs of WT and A53T mice. Scale bar, 200 μm. **B** Quantitative analysis of the Qdot 605 area in dCLNs of WT and A53T mice. *N* = 3 independent animals in each group. Values are means ± S.E.M, *t* test. **, *P* < 0.01. **Figure S2.** Increased inflammatory cytokines and ER stress in macrophages of dCLN of A53T mice. **A** Representative fluorescence images of dCLNs from WT and A53T mice treated with or without TUDCA immunolabeled by F4/80 (green) with IL-1β (red), IL-6 (red), or TNF-α (red). Scale bar, 100 μm. **B** Quantitative analysis of IL-1β, IL6 and TNF-α positive area in dCLNs of WT and A53T mice. *N* = 5 independent animals in WT and A53T. **C** Representative fluorescence images of GRP78 in macrophages (F4/80 positive) in dCLNs of WT and A53T mice. Scale bar, 200 μm. Values are means ± S.E.M, one-way ANOVA test. ns, not significant; ***, *P* < 0.001; ****, *P* < 0.0001. **Figure S3.** α-Synuclein in CSF was drained through the meningeal lymphatics. **A** Schematic image of the experimental setup for detection of lymphatic drainage path of α-synuclein in CSF. **B**, **C** Representative fluorescence images of meninges of C57 mice injected (i.c.m) with vehicle or AF647-α-synuclein (red). Scale bar, 1 mm. **D**, **E** Zoom-in images of the petrosquamous sinus in B, C. Scale bar, 200 μm. **F**, **G** Zoom-in images of the rostral rhinal vein field in B, C. Scale bar, 200 μm. **H** Western blot to detect α-synuclein in dCLNs of C57 mice intra-cisternally injected with vehicle, AF647-α-synuclein and α-synuclein. **I** Quantitative analysis of α-synuclein protein levels in dCLNs injected (i.c.m) with vehicle control, AF647-α-synuclein and α-synuclein. *N* = 3 independent animals in vehicle control and AF647-α-synuclein group, *N* = 2 independent animals in α-synuclein group. Values are means ± S.E.M, one-way ANOVA test. ns, not significant; *, *P* < 0.05. **Figure S4.** α-Synuclein in CNS was drained to the macrophage in dCLN. **A** Representative fluorescence images of dCLNs in A53T mice immunolabeled with CD11b (green) and MJFR-1 (red). Scale bar, 200 μm. **B** Zoom-in image of white dotted box in A. White arrow indicates the colocalization of CD11b and α-synuclein. Scale bar, 200 μm. **C** Representative fluorescence images of dCLNs in A53T mice immunolabeled with CD169 (green) and MJFR-1 (red). Scale bar, 200 μm. **D** Zoom-in image of white dotted box in C. White arrow indicates the colocalization of CD169 and α-synuclein. Scale bar, 200 μm. **E**, **F** Quantitative analysis of the levels (pg/mg) of total α-synuclein and α-synuclein oligomer in macrophages of dCLN and total dCLN lysate using MSD, normalized by total protein concentration. *N* = 3 independent sample pools in each group. Values are means ± S.E.M, *t* test. *, *P* < 0.05. **Figure S5.** Peripheral inflammation in A53T mice. **A** Volcano plot of the significantly up- and down-regulated genes in the dCLNs of WT and A53T mice. *N* = 3 independent in each group (3 WT samples were pooled from 6 animals; 3 A53T samples were pooled from 4 animals). *P* values (*P*_adj_, adjusted *P* values) were corrected for multiple hypothesis testing with the Benjamini–Hochberg false discovery rate procedure. **B** Principal component (PC) analysis of the transcriptome of dCLNs of WT and A53T mice. *N* = 3 independent in each group (3 WT samples were pooled from 6 animals; 3 A53T samples were pooled from 4 animals). **C** The scatter diagram of GO enrichment of differentially expressed genes in dCLNs of WT and A53T mice. **D** Quantitative analysis of the mRNA level of typical genes in ER stress, including *Ern1*, *Xbp1*, *Atf6*, *Atf4*, *Ddit3*, and *Hspa5*. *N* = 3 independent animals in each group. Values are means ± S.E.M, *t* test. *, *P* < 0.05. **Figure S6.** Heat map of genes involved in response to endoplasmic reticulum stress signaling pathway. Red represents gene expression levels above the mean; blue represents gene expression levels below the mean. *N* = 3 independent in each group (3 WT samples were pooled from 6 animals; 3 A53T samples were pooled from 4 animals). **Figure S7.** α-Synuclein monomer elicited ER stress weakly in macrophages. **A** Western blot to assess the levels of p-IRE1α, IRE1α, GRP78, ATF6, ATF6-p, ATF4, sXBP-1, p-eIF2α, eIF2α and α-synuclein in BMDMs treated with vehicle control, 100 nM or 400 nM α-synuclein monomer. **B**–**J** Quantitative analysis of the levels of p-IRE1α, IRE1α, RP78, ATF6, ATF6-p, ATF4, sXBP-1, p-eIF2α and eIF2α in BMDMs treated with vehicle control, 100 nM or 400 nM α-synuclein monomer. *N* = 3 independent experiments in each group. **K** Quantitative analysis of *Ddit-3* mRNA levels in BMDMs treated with vehicle control, 100 nM or 400 nM α-synuclein monomer using qPCR. *N* = 3 independent experiments in each group. Values are means ± S.E.M, one-way ANOVA test. ns, not significant; *, *P* < 0.05; **, *P* < 0.01; ***, *P* < 0.001. **Figure S8.** α-Synuclein aggregate elicited ER stress in the dCLN. **A** Schematic image of the experimental setup to verify whether α-synuclein oligomer can elicit ER stress in the dCLN. **B** Western blot to assess the levels of p-IRE1α, IRE1α, GRP78, ATF6, ATF6-p, p-eIF2α, and eIF2α in dCLNs of C57 mice treated with vehicle control or 2 μg α-synuclein oligomer. **C**–**I** Quantitative analysis of the levels of p-IRE1α, IRE1α, GRP78, ATF6, ATF6-p, p-eIF2α and eIF2α in dCLNs of C57 mice treated with vehicle control or 2 μg α-synuclein oligomer. *N* = 3 independent experiments in each group (3 samples were pooled from 6 animals). Values are means ± S.E.M, one-way ANOVA test. ns, not significant; *, *P* < 0.05; **, *P* < 0.01. **Figure S9.** TUDCA improved the motor function and rescued neuronal death of A53T mice. **A** The latency to fall off from the rod for WT and A53T mice treated with or without TUDCA. *N* = 5 independent animals in each group. **B** The time taken for the mice to land from the top for WT and A53T mice treated with or without TUDCA in the pole test. *N* = 5 independent animals in each group. **C**, **D** Immunohistochemical staining and quantification of tyrosine hydroxylase (TH) positive neurons in the substantia nigra of WT and A53T mice treated with or without TUDCA. *N* = 3 independent animals in each group. Values are means ± S.E.M, one-way ANOVA test. ns, not significant; *, *P* < 0.05; **, *P* < 0.01; ***, *P* < 0.005, ****, *P* < 0.001. **Table S1.** Numbers of animals per study group.

**Additional file 2: Table S2.** Differential expression of genes between dCLNs of WT and A53T mice.

**Additional file 3: Video S1.** The flow of Qdot 605 in dCLNs of WT mice.

**Additional file 4: Video S2.** The flow of Qdot 605 in dCLNs of A53T mice.

## Data Availability

All data generated or analyzed during this study are included in this published article and its Additional files.

## Abbreviations

PD: Parkinson’s disease
α-syn: α-Synuclein
CNS: Central nervous system
dCLN: Deep cervical lymph node
CSF: Cerebrospinal fluid
BBB: Blood-brain barrier
ER stress: Endoplasmic reticulum stress
UPR: Unfolded protein response
TUDCA: Tauroursodeoxycholic acid
BMDM: Bone-marrow-derived macrophage
SCS: Subcapsular sinus
