# Supplementary material for: The cervical lymph node contributes to peripheral inflammation related to Parkinson’s disease
Source: J Neuroinflammation. 2023 Apr 10;20:93. doi: 10.1186/s12974-023-02770-5 (PMC10088204; doi:10.1186/s12974-023-02770-5)
Supplement: Supplementary file 1 — Additional file 1: Figure S1. Distribution of Qdot605 in dCLNs of A53T mice. A Representative fluorescence images of dCLNs of WT and A53T mice. Scale bar, 200 μm. B Quantitative analysis of the Qdot 605 area in dCLNs of WT and A53T mice. N = 3 independent animals in each group. Values are means ± S.E.M, t test. **, P < 0.01. Figure S2. Increased inflammatory cytokines and ER stress in macrophages of dCLN of A53T mice. A Representative fluorescence images of dCLNs from WT and A53T mice treated with or without TUDCA immunolabeled by F4/80 (green) with IL-1β (red), IL-6 (red), or TNF-α (red). Scale bar, 100 μm. B Quantitative analysis of IL-1β, IL6 and TNF-α positive area in dCLNs of WT and A53T mice. N = 5 independent animals in WT and A53T. C Representative fluorescence images of GRP78 in macrophages (F4/80 positive) in dCLNs of WT and A53T mice. Scale bar, 200 μm. Values are means ± S.E.M, one-way ANOVA test. ns, not significant; ***, P < 0.001; ****, P < 0.0001. Figure S3. α-Synuclein in CSF was drained through the meningeal lymphatics. A Schematic image of the experimental setup for detection of lymphatic drainage path of α-synuclein in CSF. B, C Representative fluorescence images of meninges of C57 mice injected (i.c.m) with vehicle or AF647-α-synuclein (red). Scale bar, 1 mm. D, E Zoom-in images of the petrosquamous sinus in B, C. Scale bar, 200 μm. F, G Zoom-in images of the rostral rhinal vein field in B, C. Scale bar, 200 μm. H Western blot to detect α-synuclein in dCLNs of C57 mice intra-cisternally injected with vehicle, AF647-α-synuclein and α-synuclein. I Quantitative analysis of α-synuclein protein levels in dCLNs injected (i.c.m) with vehicle control, AF647-α-synuclein and α-synuclein. N = 3 independent animals in vehicle control and AF647-α-synuclein group, N = 2 independent animals in α-synuclein group. Values are means ± S.E.M, one-way ANOVA test. ns, not significant; *, P < 0.05. Figure S4. α-Synuclein in CNS was drained to the macrophage in dCLN. A [file 12974_2023_2770_MOESM1_ESM.docx]

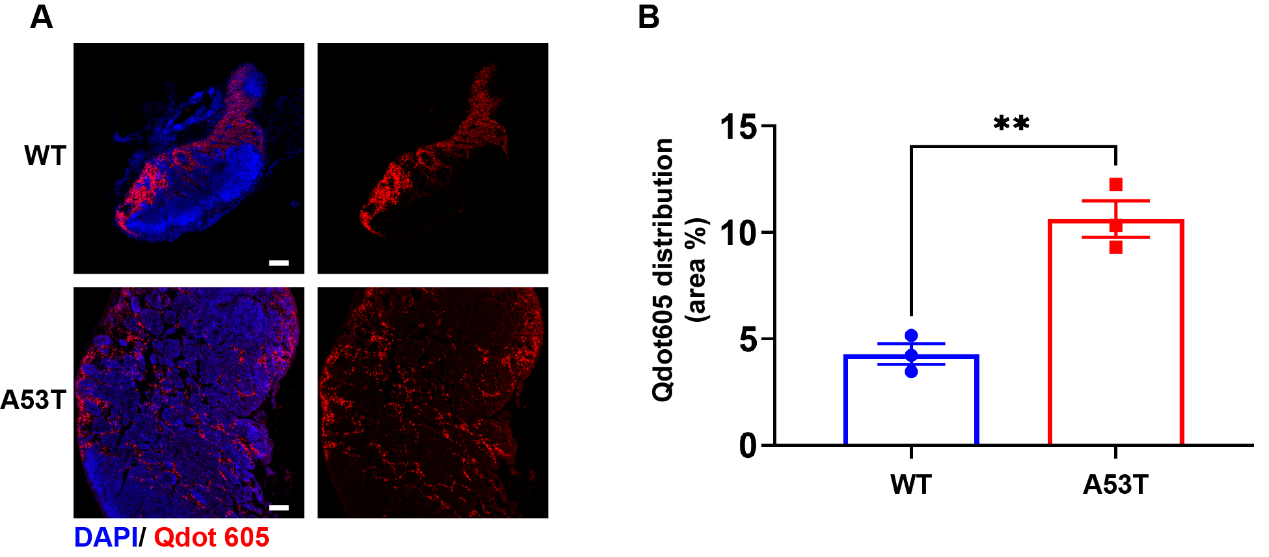
**Figure S1. Distribution of Qdot605 in dCLNs of A53T mice.** (**A**) Representative fluorescence images of dCLNs of WT and A53T mice. Scale bar, 200 μm. (**B**) Quantitative analysis of the Qdot 605 area in dCLNs of WT and A53T mice. N = 3 independent animals in each group. Values are means ± S.E.M, t test. **, *P* < 0.01.


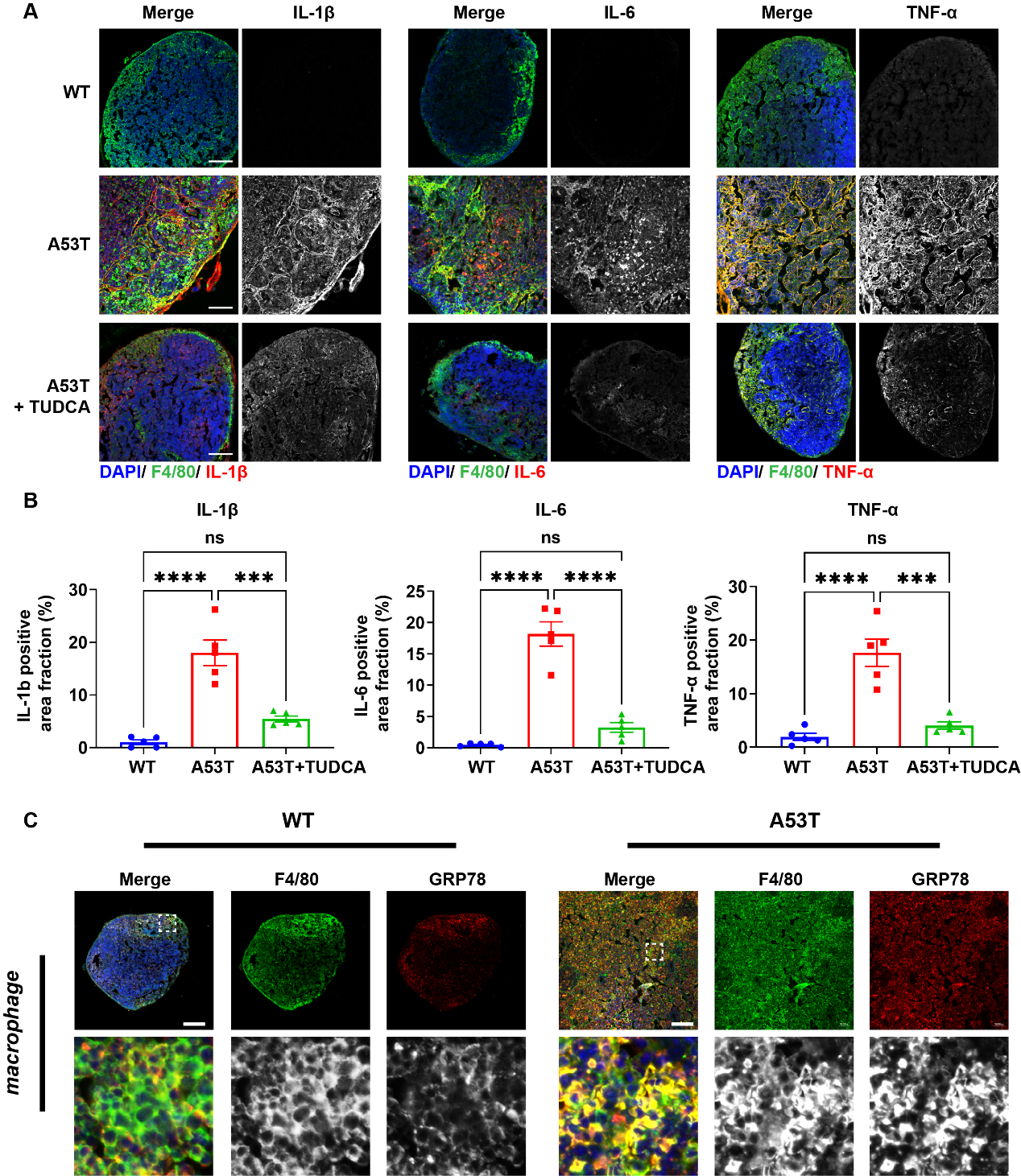


**Figure S2. Increased inflammatory cytokines and ER stress in macrophages of dCLN of A53T mice. (A)** Representative fluorescence images of dCLNs from WT and A53T mice treated with or without TUDCA immunolabeled by F4/80 (green) with IL-1β (red), IL-6 (red), or TNF-α (red). Scale bar, 100 μm. (**B**) Quantitative analysis of IL-1β, IL6 and TNF-α positive area in dCLNs of WT and A53T mice. N = 5 independent animals in WT and A53T. (**C**) Representative fluorescence images of GRP78 in macrophages (F4/80 positive) in dCLNs of WT and A53T mice. Scale bar, 200 μm. Values are means ± S.E.M, one-way ANOVA test. ns, not significant; ***, *P* < 0.001; ****, *P* < 0.0001.


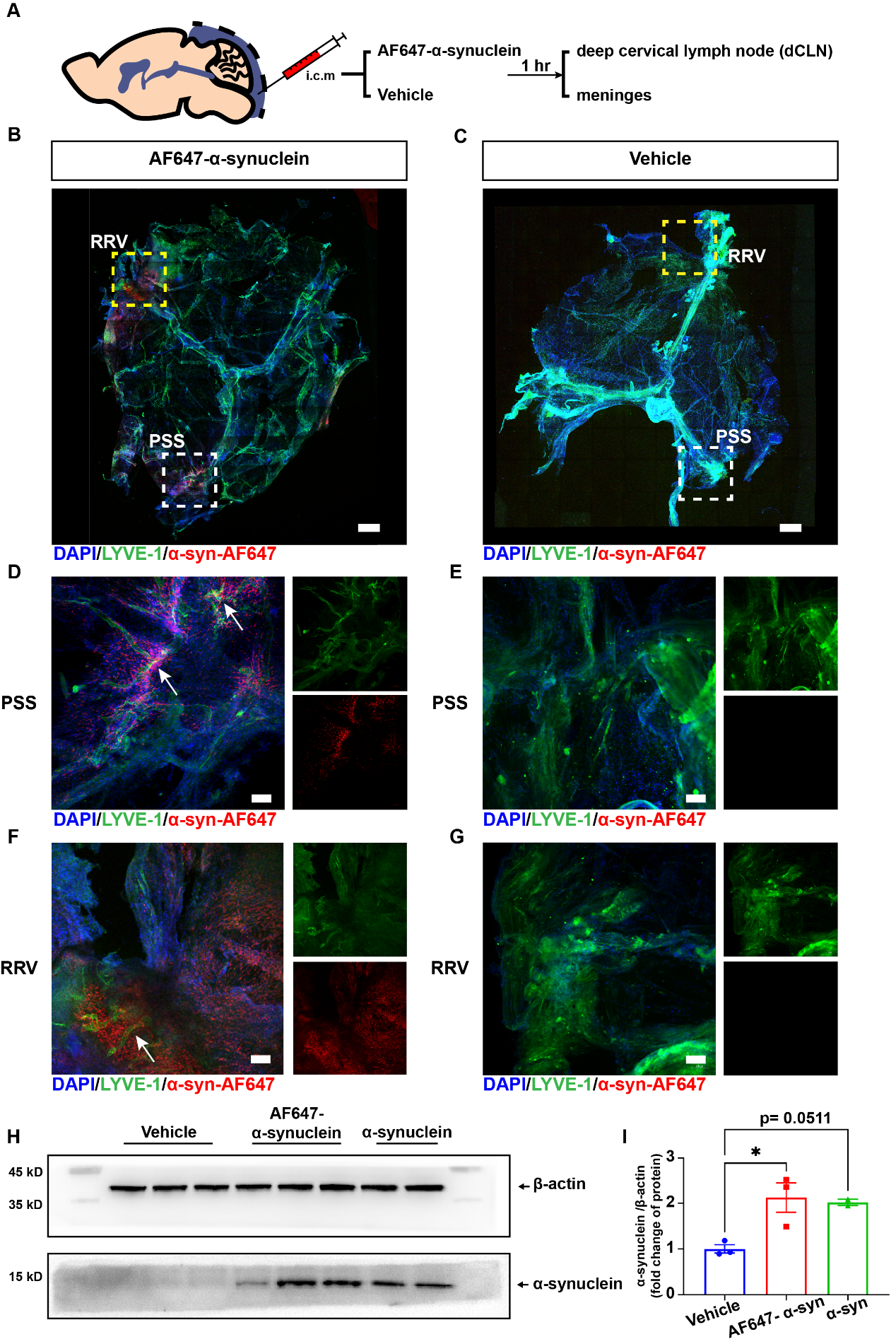


**Figure S3. α-synuclein in CSF was drained through the meningeal lymphatics.** (**A**) Schematic image of the experimental setup for detection of lymphatic drainage path of α-synuclein in CSF. (**B, C**) Representative fluorescence images of meninges of C57 mice injected (i.c.m) with vehicle or AF647-α-synuclein (red). Scale bar, 1 mm. (**D, E**) Zoom-in images of the petrosquamous sinus in B, C. Scale bar, 200 μm. (**F-G**) Zoom-in images of the rostral rhinal vein field in B, C. Scale bar, 200 μm. (**H**) Western blot to detect α-synuclein in dCLNs of C57 mice intra-cisternally injected with vehicle, AF647-α-synuclein and α-synuclein. (**I**) Quantitative analysis of α-synuclein protein levels in dCLNs injected (i.c.m) with vehicle control, AF647-α-synuclein and α-synuclein. N = 3 independent animals in vehicle control and AF647-α-synuclein group, N = 2 independent animals in α-synuclein group. Values are means ± S.E.M, one-way ANOVA test. ns, not significant; *, *P* < 0.05.


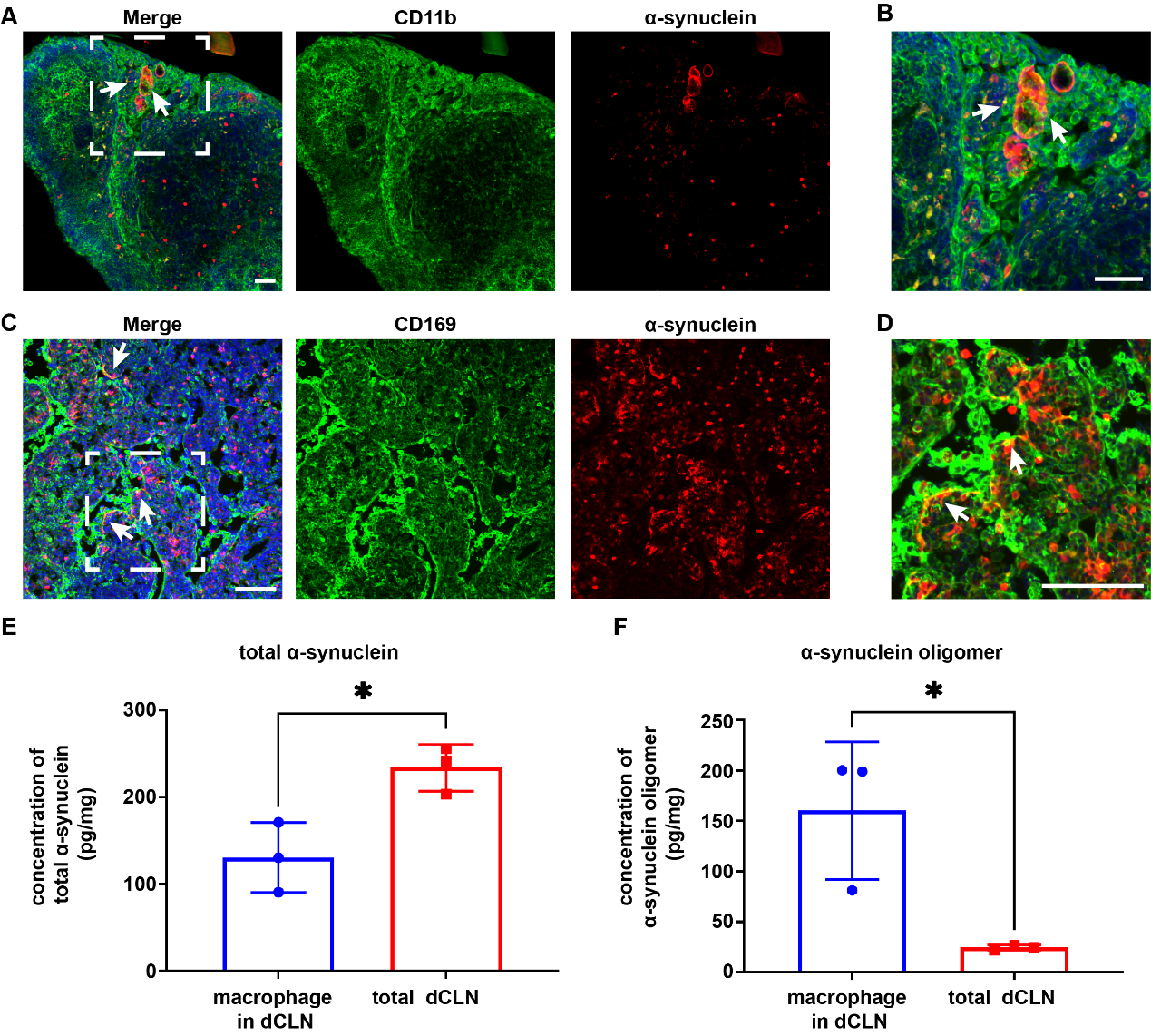
 **Figure S4. α-synuclein in CNS was drained to the macrophage in dCLN.** (**A**) Representative fluorescence images of dCLNs in A53T mice immunolabeled with CD11b (green) and MJFR-1 (red). Scale bar, 200 μm. **(B)** Zoom-in image of white dotted box in A. White arrow indicates the colocalization of CD11b and α-synuclein. Scale bar, 200 μm. **(C)** Representative fluorescence images of dCLNs in A53T mice immunolabeled with CD169 (green) and MJFR-1 (red). Scale bar, 200 μm. **(D)** Zoom-in image of white dotted box in C. White arrow indicates the colocalization of CD169 and α-synuclein. Scale bar, 200 μm. (**E, F**) Quantitative analysis of the levels (pg/mg) of total α-synuclein and α-synuclein oligomer in macrophages of dCLN and total dCLN lysate using MSD, normalized by total protein concentration. N = 3 independent sample pools in each group. Values are means ± S.E.M, t test. *, *P* < 0.05.


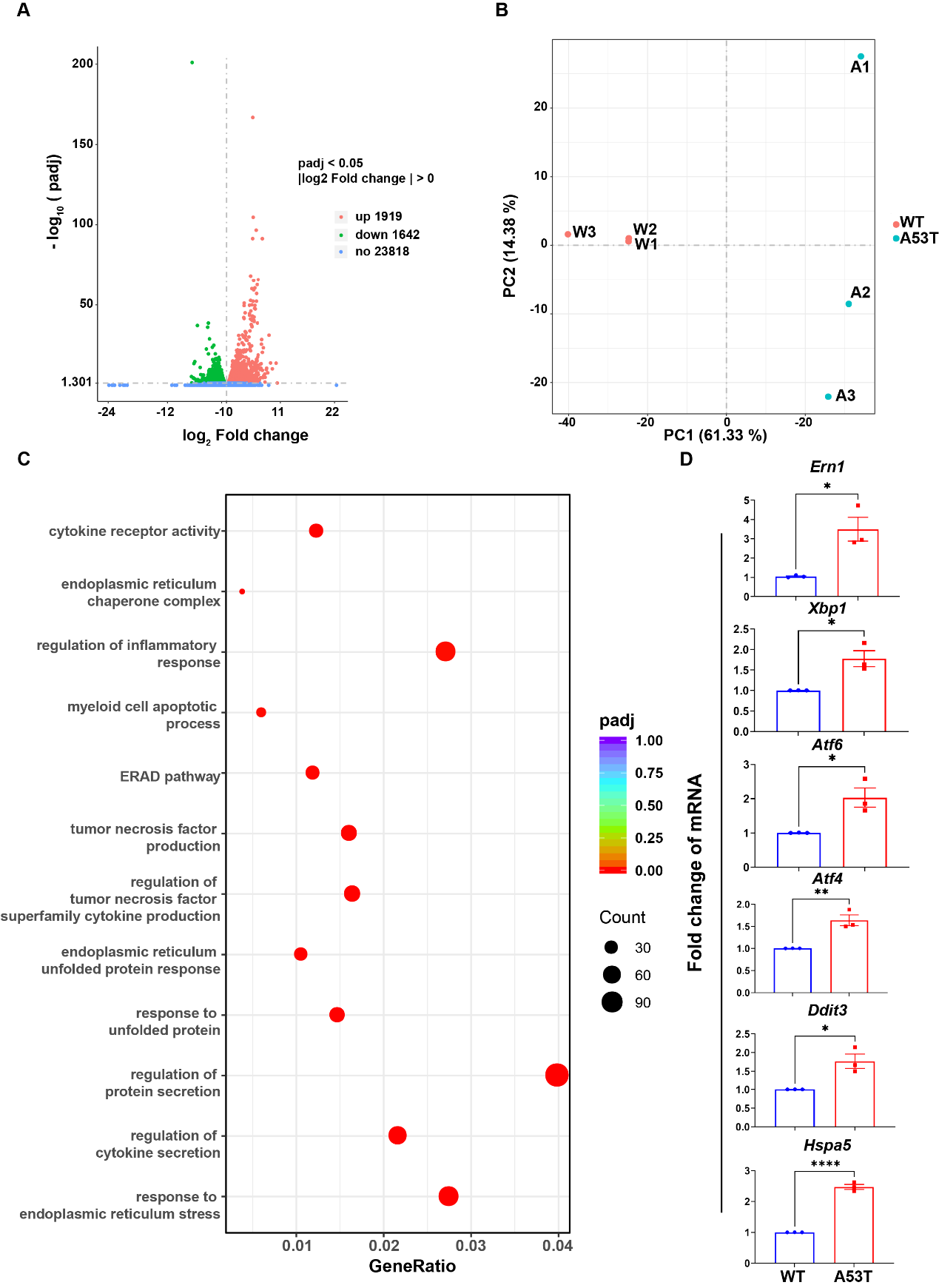


**Figure S5. Peripheral inflammation in A53T mice.** (**A**) Volcano plot of the significantly up- and down-regulated genes in the dCLNs of WT and A53T mice. N = 3 independent in each group (3 WT samples were pooled from 6 animals; 3 A53T samples were pooled from 4 animals). P values (Padj, adjusted P values) were corrected for multiple hypothesis testing with the Benjamini–Hochberg false discovery rate procedure. (**B**) Principal component (PC) analysis of the transcriptome of dCLNs of WT and A53T mice. N = 3 independent in each group (3 WT samples were pooled from 6 animals; 3 A53T samples were pooled from 4 animals). (**C**) The scatter diagram of GO enrichment of differentially expressed genes in dCLNs of WT and A53T mice. (**D**) Quantitative analysis of the mRNA level of typical genes in ER stress, including *Ern1*, *Xbp1*, *Atf6*, *Atf4*, *Ddit3*, and *Hspa5*. N = 3 independent animals in each group. Values are means ± S.E.M, t test. *, P < 0.05.


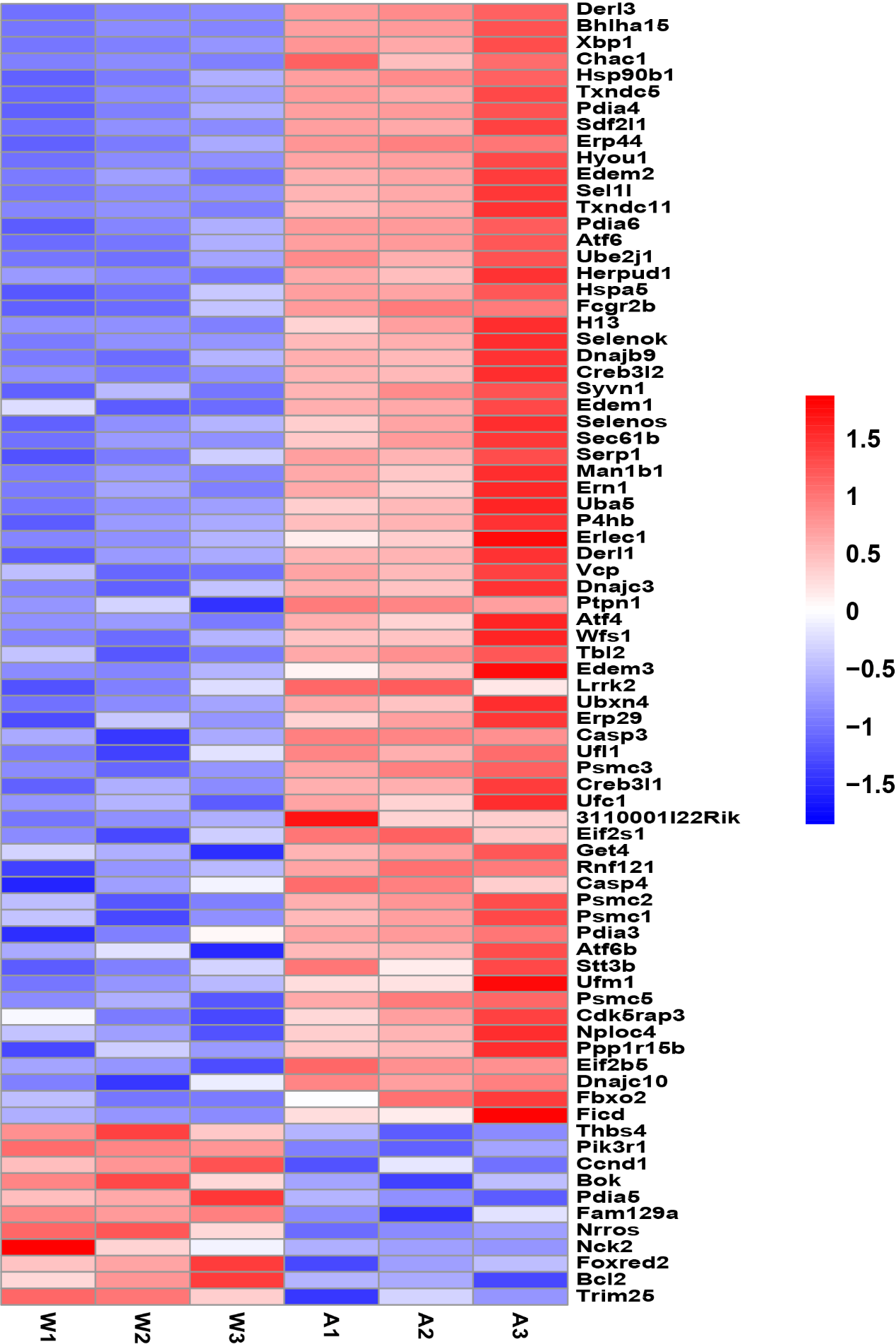


**Figure S6. Heat map of genes involved in response to endoplasmic reticulum stress signaling pathway.** Red represents gene expression levels above the mean; blue represents gene expression levels below the mean. N = 3 independent in each group (3 WT samples were pooled from 6 animals;3 A53T samples were pooled from 4 animals).


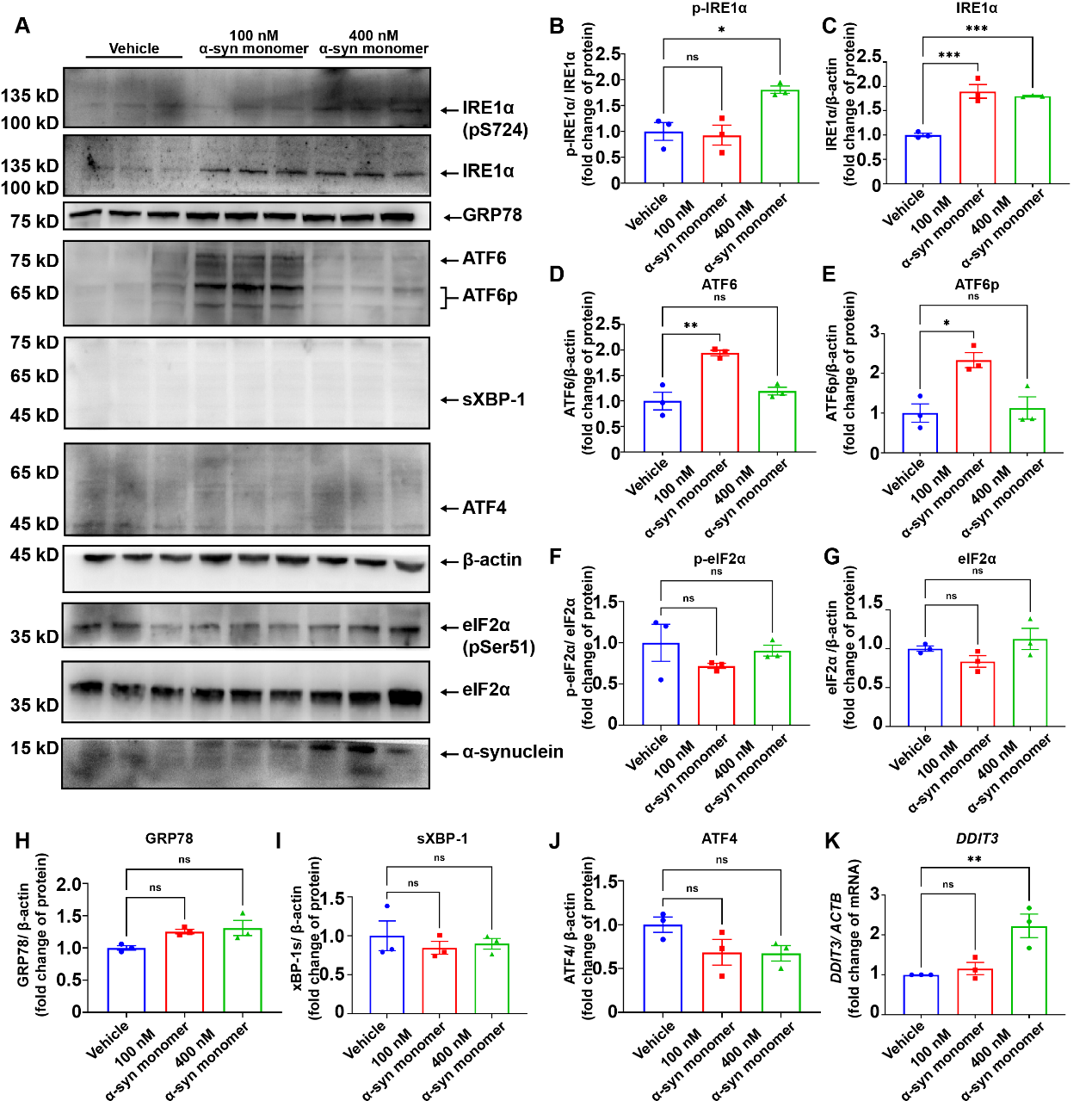


**Figure S7. α-synuclein monomer elicited ER stress weakly in macrophages.** (**A**) Western blot to assess the levels of p-IRE1α, IRE1α, GRP78, ATF6, ATF6-p, ATF4, sXBP-1, p-eIF2α, eIF2α and α-synuclein in BMDMs treated with vehicle control, 100 nM or 400 nM α-synuclein monomer. **(B-J)** Quantitative analysis of the levels of p-IRE1α, IRE1α, GRP78, ATF6, ATF6-p, ATF4, sXBP-1, p-eIF2α and eIF2α in BMDMs treated with vehicle control, 100 nM or 400 nM α-synuclein monomer. N = 3 independent experiments in each group. (**K**) Quantitative analysis of *Ddit-3* mRNA levels in BMDMs treated with vehicle control, 100 nM or 400 nM α-synuclein monomer using qPCR. N = 3 independent experiments in each group. Values are means ± S.E.M, one-way ANOVA test. ns, not significant; *, *P* < 0.05; **, *P* < 0.01; ***, *P* < 0.001.


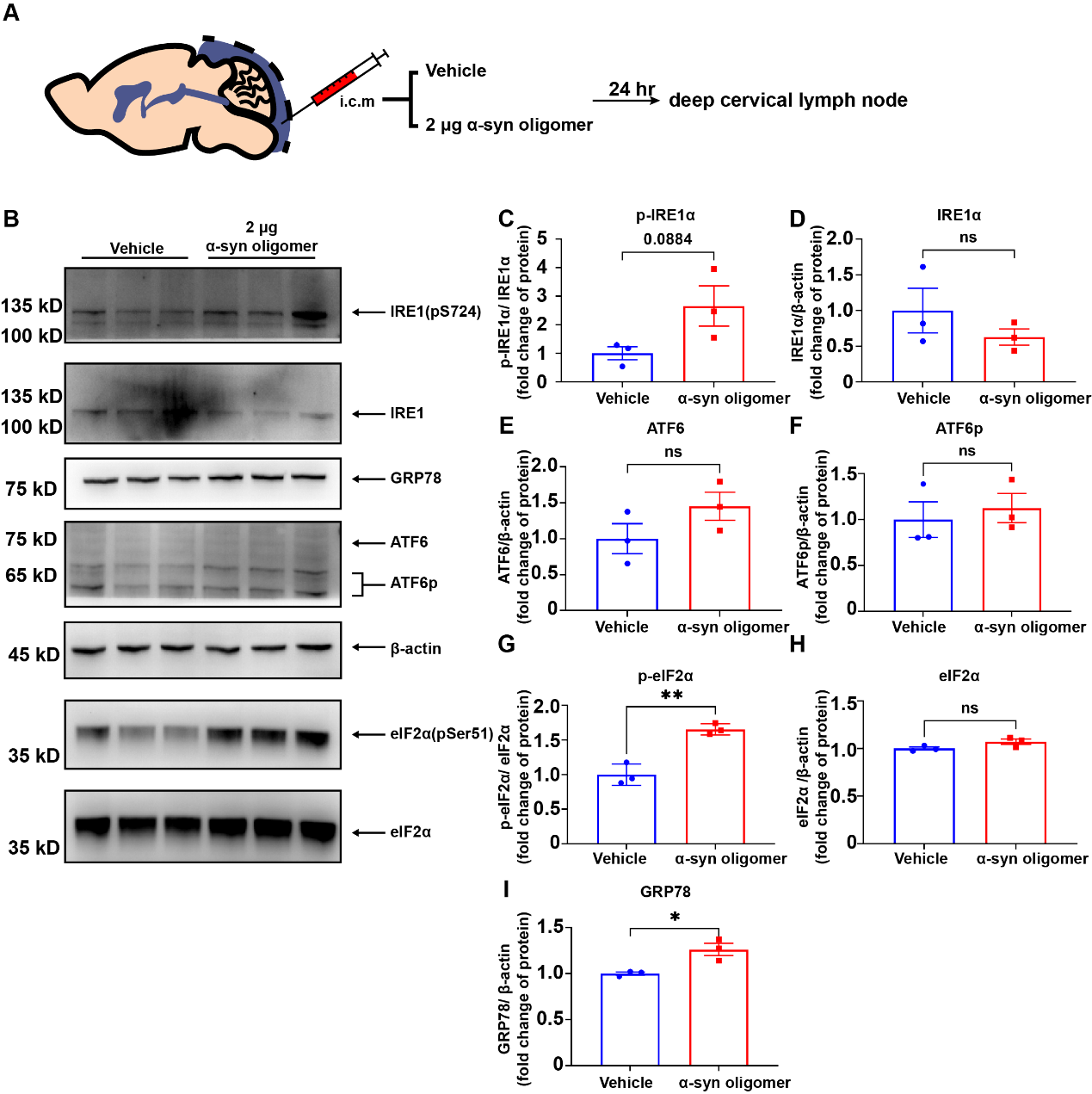


**Figure S8.** **α-synuclein aggregate elicited ER stress in the dCLN.** (**A**) Schematic image of the experimental setup to verify whether α-synuclein oligomer can elicit ER stress in the dCLN. (**B**) Western blot to assess the levels of p-IRE1α, IRE1α, GRP78, ATF6, ATF6-p, p-eIF2α, and eIF2α in dCLNs of C57 mice treated with vehicle control or 2 μg α-synuclein oligomer. **(C-I)** Quantitative analysis of the levels of p-IRE1α, IRE1α, GRP78, ATF6, ATF6-p, p-eIF2α and eIF2α in dCLNs of C57 mice treated with vehicle control or 2 μg α-synuclein oligomer. N = 3 independent experiments in each group (3 samples were pooled from 6 animals). Values are means ± S.E.M, one-way ANOVA test. ns, not significant; *, P < 0.05; **, P < 0.01.


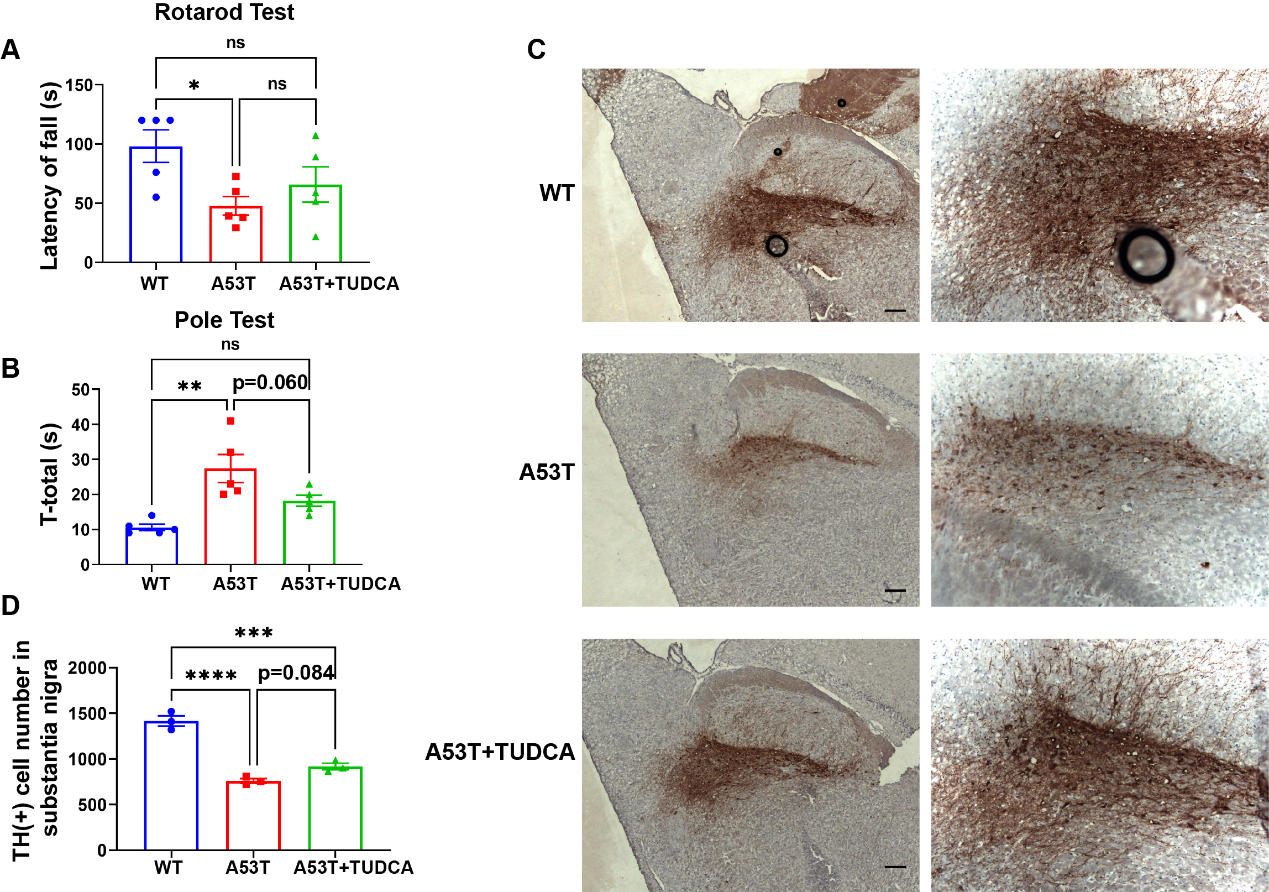


**Figure S9. TUDCA improved the motor function and rescued neuronal death of A53T mice.** (**A**) The latency to fall off from the rod for WT and A53T mice treated with or without TUDCA. N = 5 independent animals in each group. (**B**) The time taken for the mice to land from the top for WT and A53T mice treated with or without TUDCA in the pole test. N = 5 independent animals in each group. (**C-D**) Immunohistochemical staining and quantification of tyrosine hydroxylase (TH) positive neurons in the substantia nigra of WT and A53T mice treated with or without TUDCA. N = 3 independent animals in each group. Values are means ± S.E.M, one-way ANOVA test. ns, not significant; *, P < 0.05; **, P < 0.01; ***, P < 0.005, ****, P < 0.001.

**Table S1 Numbers of animals per study group**

| Experiment/genotype | WT (B6C3F1/J) | A53T | C57Bl/6J |
| --- | --- | --- | --- |
| immunohistochemistry | 14 | 16 | 0 |
| Intravital Microscopy | 4 | 4 | 0 |
| qPCR | 3 | 3 | 0 |
| WB | 14 | 10 | 0 |
| Flow cytometry | 0 | 0 | 3 |
| FACS | 0 | 6 | 0 |
| i.c.m AF647-α-syn | 0 | 0 | 6 |
| i.c.m α-syn | 0 | 0 | 2 |
| i.c.m oligomeric α-syn | 0 | 0 | 6 |
| i.c.m vehicle | 0 | 0 | 9 |
| RNA-seq | 6 | 4 | 0 |
| TUDCA treatment | 7/7 | 6/6 | 0 |
| Vehicle treatment | 7/7 | 6/5 (one died during treatment) | 0 |
